# Optical suppression of energy barriers in single molecule-metal binding

**DOI:** 10.1126/sciadv.abp9285

**Published:** 2022-06-24

**Authors:** Qianqi Lin, Shu Hu, Tamás Földes, Junyang Huang, Demelza Wright, Jack Griffiths, Eoin Elliott, Bart de Nijs, Edina Rosta, Jeremy J. Baumberg

**Affiliations:** 1 Nanophotonics Centre, Department of Physics, Cavendish Laboratory, University of Cambridge, Cambridge CB3 0HE, England, UK.; 2 Department of Chemistry, King’s College London, 7 Trinity Street, London SE1 1DB, UK.; 3 Department of Physics and Astronomy, University College London, London WC1E 6BT, UK.

## Abstract

Transient bonds between molecules and metal surfaces underpin catalysis, bio/molecular sensing, molecular electronics, and electrochemistry. Techniques aiming to characterize these bonds often yield conflicting conclusions, while single-molecule probes are scarce. A promising prospect confines light inside metal nanogaps to elicit in operando vibrational signatures through surface-enhanced Raman scattering. Here, we show through analysis of more than a million spectra that light irradiation of only a few microwatts on molecules at gold facets is sufficient to overcome the metallic bonds between individual gold atoms and pull them out to form coordination complexes. Depending on the molecule, these light-extracted adatoms persist for minutes under ambient conditions. Tracking their power-dependent formation and decay suggests that tightly trapped light transiently reduces energy barriers at the metal surface. This opens intriguing prospects for photocatalysis and controllable low-energy quantum devices such as single-atom optical switches.

## Teaser

Light trapped by single metal atoms is shown to generate huge optical forces that can pull them out of metal surfaces.

## INTRODUCTION

Understanding the interactions between molecules and metal surfaces is of widespread importance in both fundamental science and evolving technologies, with prominent examples spanning electrochemistry (*1*), catalysis (*2*), organic solar cells (*3*), biosensing (*4*), and medical imaging/targeting (*5*). In particular, there has been tremendous interest in using molecule-metal transient bonds for the development of molecular electronics (*6*–*8*) and spintronics (*9*–*11*) to enable ultrahigh-density information devices for low-energy and compact upscaled data storage.

To study molecule-metal surface interactions, spectroscopic techniques such as Raman or infrared absorption have great utility since they work in ambient conditions, are nondestructive, and can track dynamics, resolve transient species, and work in electrochemical environments (*12*). To avoid problematic averaging over many different sites and conformations, the confinement of optical fields to atomic scales has enabled vibrational spectroscopy of single molecules at the metal interface. Plasmonic field localization to picometer length scales on metals (termed picocavities) depends on individual metal adatoms, as described by recent theories (*13*–*15*). Tip-enhanced (*16*–*18*) and surface-enhanced (*19*–*22*) Raman spectroscopies (TERS and SERS) now reach this picocavity regime.

Extensive experiments and modeling of adatoms using scanning tunneling microscopy (often in ultrahigh vacuum at cryogenic temperatures) have shown typical $U_{f}^{0}\sim$1 eV adatom formation energies (for coinage metals), as well as ~1 eV surface diffusion barriers (*23*–*27*), both key energy scales in catalysis. However, how these change with molecule-metal interactions or with light and how they link to picocavity formation in metal nanogaps are not understood (*28*). This is exacerbated by the challenge of unifying quantum molecular modeling with classical electromagnetism on these atomic length scales (*29*).

In this work, we demonstrate how the molecule-metal opto-chemical interaction directly influences the formation and stabilization of light-induced adatoms yielding picocavities. Previously, we showed how picocavities give vibrational spectra from single molecules (*19*, *21*) but were unable to shed light on the mechanisms for how they are formed. Here, we show that power-dependent creation rates depend crucially on the molecular species in their vicinity, a key constraint to any proposed mechanism. We emphasize that while the temperature of the metal remains near room temperature (evidenced directly in the SERS), optical heating would instead require $T>U_{f}^{0}/k_{B}\sim 12,000\;K$ to overcome the adatom formation barrier $U_{f}^{0}\sim 1$ eV and extract a single Au^(0)^ atom from the gold surface. After presenting the data, we discuss what models might account for the observations. Our results suggest the mechanism to be opto-molecular tuning of the energy barrier at metal surfaces, which opens up opportunities for controlling reactions at the single-atom and single-molecule level, with promise in catalysis, optical switching, quantum devices, and molecular optoelectronics.

## RESULTS

### Plasmonic nanocavity assembly

**Fig. 1.**
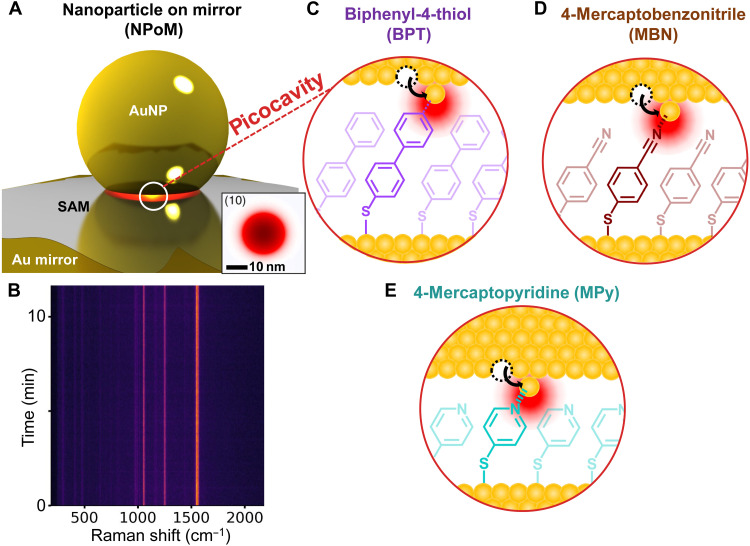
Light-activated molecular control of adatoms. (**A**) NPoM geometry, sandwiching a SAM of either BPT (purple), MBN (brown), or MPy (cyan). Optical field at plasmon resonance is trapped in the nanogap (inset shows cross section under facet through the gap center). (**B**) SERS spectra from BPT NPoM every 100 ms at 20 μW, showing no picocavities at low power. (**C** to **E**) Picocavities formed by a single adatom protrusion.

To confine the light sufficiently tightly, we construct nanocavities from nanoparticle-on-mirror (NPoM) plasmonic gaps (Fig. 1A) that can incorporate a wide range of molecules. A self-assembled monolayer (SAM) is immobilized onto planar gold ‘mirror’ substrates with gold nanoparticles (AuNPs) and then drop-cast on top. We focus on monolayers of biphenyl-4-thiol (BPT; purple in Fig. 1), 4-mercaptobenzonitrile (MBN; brown), and 4-mercaptopyridine (MPy; cyan) sandwiched inside the gaps but emphasize that most molecules give similar results (others in fig. S1) (*21*, *30*). The dipolar coupling between the nanoparticle and its image charges in the mirror creates a strong electromagnetic field tightly confined in the nanoscale hotspot between the facets (Fig. 1A, inset), enhancing Raman scattering by >10^9^. The properties of the monolayer can be characterized by dark-field scattering spectra of the NPoMs giving their nanocavity resonant wavelengths (λ*_c_*) (fig. S2) (*31*). Irradiating NPoMs with a laser at 633 nm gives efficiently outcoupled SERS spectra suited for rapid spectral acquisition at subsecond rates with powers down to 1 μW, without substantially heating them (see Materials and Methods). At low powers, no picocavities are observed (Fig. 1B).

### Adatom movement

**Fig. 2.**
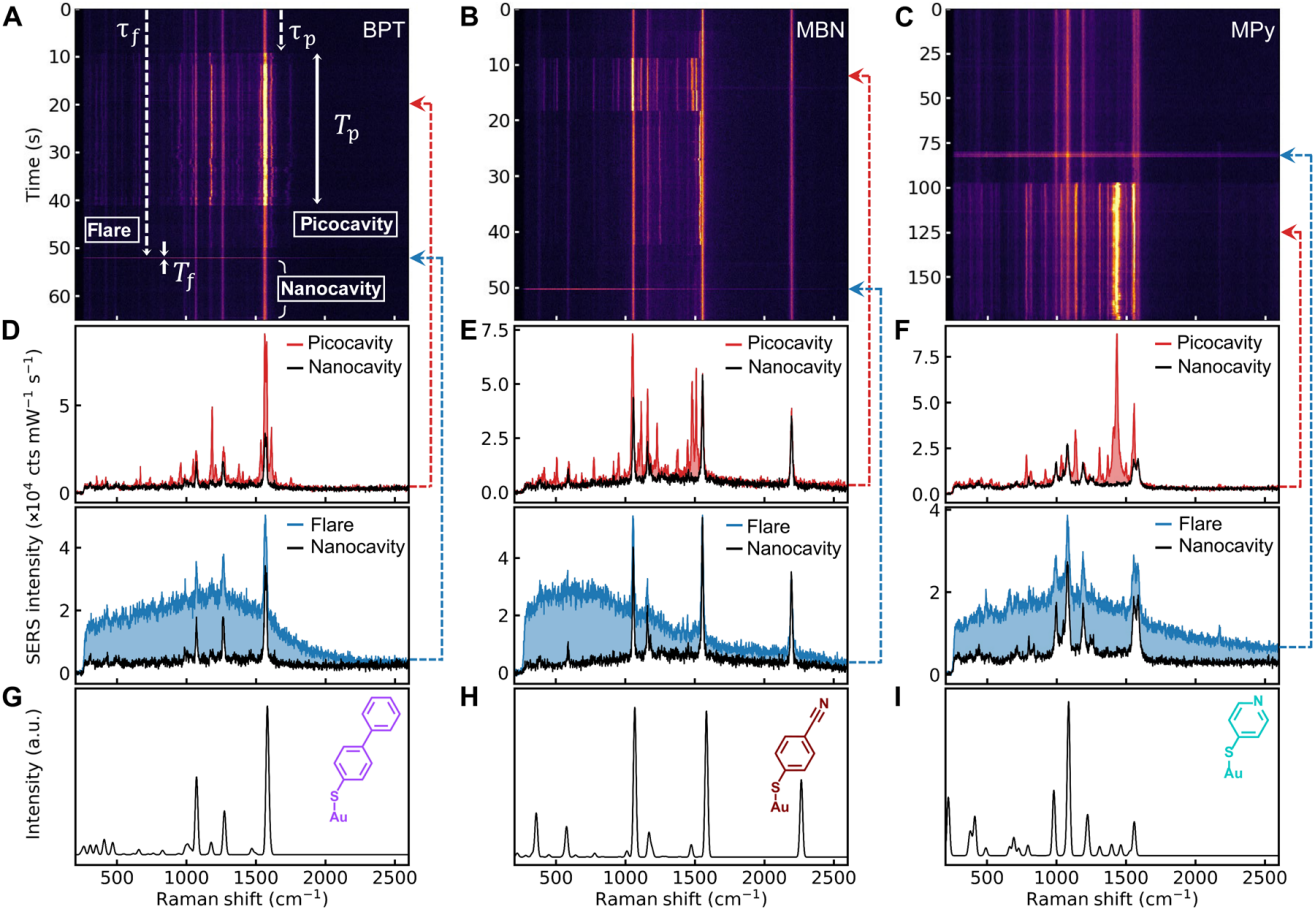
Raman time evolution of picocavities and flares. (**A**) Time-series SERS spectra of BPT for 50-μW 633-nm laser irradiation. Examples of a nanocavity, a picocavity, and a flare are shown: τ_p_ is the formation time before a picocavity is observed, *T*_p_ is the lifetime of a picocavity, and τ_f_ and *T*_f_ are the formation time and lifetime of a flare. (**B**) Time-series SERS spectra of MBN for 50-μW irradiation. (**C**) Time-series SERS spectra of MPy for 10-μW irradiation. (**D** to **F**) Example SERS spectra from the nanocavity (black, *t* = 0 s), a picocavity (red), and a flare (blue). (**G** to **I**) DFT-calculated Raman spectra with the corresponding molecular structures in the insets. a.u., arbitrary units.

In SERS time series (Figs. 2, A to C, and 1B), persistent lines with constant intensity originate from the few hundred molecules located within the nanocavity hotspot (black spectra in Fig. 2, D to F) (*28*). Their identity is confirmed by comparison with density functional theory (DFT) calculations (Fig. 2, G to I; see Materials and Methods). Upon strong enough illumination, two types of atomic surface reconstructions are seen to occur at the surrounding metal facets. In the first type, picocavities (*19*, *21*, *22*) arise from single Au^(0)^ atoms pulled out of the gold surface (Fig. 1, C to E). Threefold larger field enhancements at the protruding adatom (compared to the nanocavity) give strong enough SERS (∝ 3^4^~80) to see a single molecule nearby (red, Fig. 2, D to F) above that of all other hotspot molecules together. Picocavity spectra show intense new vibrational modes that vary in both intensity and frequency through fluctuations in the single adatom-molecule coordination bond that is shown to form (*21*), as seen in DFT simulations (fig. S3) (*21*) [the vibrational energy fluctuations evidence single molecules, vibrations downshift as coordination bonds capture electron density (*32*), and selection rules break in the high field gradients]. While, very occasionally, two molecules appear with correlated vibrational fluctuations, almost always, only a single picocavity molecule is observed, hence the conclusion that only single Au adatoms are involved for each. Experiments using different laser wavelengths show that these picocavities are predominantly located away from the facet edges (*33*), near the facet center where the optical fields are strongest (Fig. 1A, inset).

**Fig. 3.**
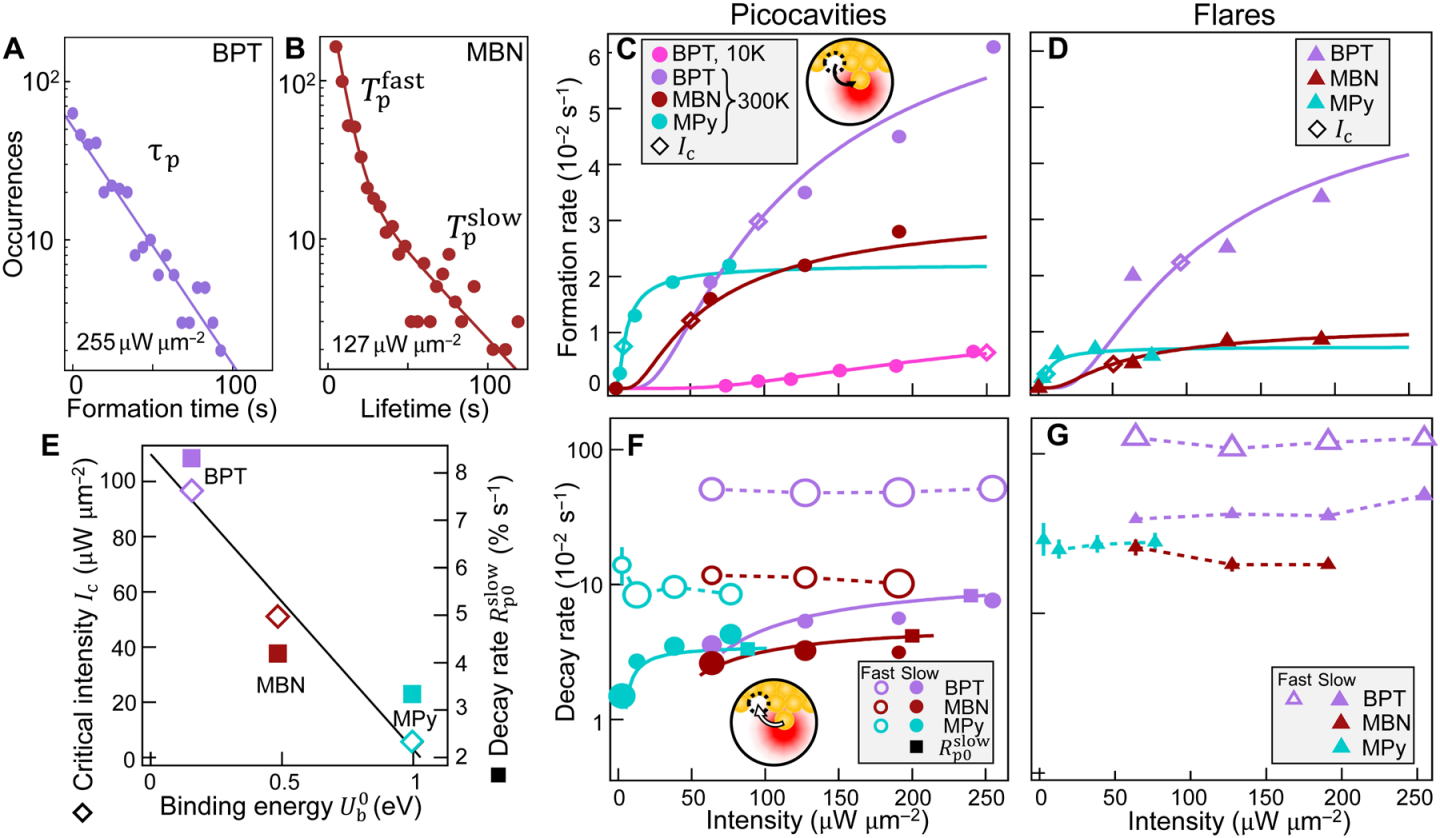
Formation and decay of picocavities and flares versus laser intensity. (**A**) Example histogram of formation times and (**B**) lifetimes of picocavities (log scale). (**C**) Formation rate of picocavities *P*_p_ at room and cryogenic temperatures, solid lines are the fits (see the main text; ◇ marks critical intensities). (**D**) Formation rate of flares *P*_f_. (**E**) Critical laser intensity *I*_c_ required for picocavity and flare formation [◇ from (C) and (D)] and saturation decay rate of slow picocavities $R_{p0}^{\text{slow}}$ [◼ from (F)] versus molecule-metal binding energy $U_{b}^{0}$ (see note S5, fig. S11, and table S6). (**F**) Decay rate of picocavities *R*_p_ (log scale), split into two classes observed as in (B): fast (open circles, <10 s for BPT and <25 s for MBN and MPy) and slow (filled circles) lifetimes [fit lines from (C)]. (**G**) Decay rate of flares *R*_f_, with fast (open triangles, <2 s) and slow (filled triangles) lifetimes. In (F) and (G), marker size gives the relative fraction of fast and slow events at each intensity. For BPT, 804,500 spectra are recorded in 1609 time series from 400 NPoMs; for MBN, 42,840 spectra in 357 time series from 119 NPoMs; and for MPy, 42,480 spectra in 236 time series from 59 NPoMs.

In the second type, ‘flares’ (*30*) arise from a region of the facet behaving differently from the bulk material. Their origin has been suggested as enhanced optical field penetration into the metal in these patches, increasing the electronic Raman scattering (ERS) (*30*); however, alternative explanations remain possible (*34*). These intense flares have broad spectra (blue, Fig. 2, D to F) that are different each time, but independent of molecule species. The lifetimes of picocavities and flares (*T*_p,f_, respectively; Fig. 2A) can be measured once formed, while statistics of their formation times (τ_p,f_) after the start of irradiation (or their average number per unit time) give their excitation rates (Fig. 3).

### Effect of light, molecule type, and temperature

#### Formation rates

To understand the opto-molecular–induced effects on picocavities and flares, thousands of time series of SERS spectra are recorded by running automated measurements using NPoM tracking software (*35*), with power-series laser irradiation from 2 to 200 μW on many different NPoMs across each sample (Fig. 3). This enables conclusions to be drawn without depending on the specific nanoparticle (near-spherical) shape and facet. Within a time series, the spectral dissimilarity metric of their Euclidean distance (*36*) and a supervised machine learning model (fig. S5) are used to discern picocavities and flares. This analysis generates statistics (table S1 to S4) that identify formation and decay rates. Exponential probability distributions are seen in all cases (Fig. 3, A and B), suggesting that a single formation process is likely responsible (notes S3 and S4).

The formation rate of picocavities (*P*_p_; Fig. 3C) initially rises slowly with laser intensity (*I*), increasing rapidly above an intensity threshold, before saturating at *P*_0p_. This is seen across all molecules measured [at *T* = 300 K, and for BPT, at 10 K (*19*); tables S1 and S3]. The critical intensity threshold *I*_c_ taken as *P*_p_(*I*_c_) = *e*^−1^*P*_0p_ is found at *I*_c_ = 97, 51, and 5.5 μW μm^−2^ for BPT, MBN, and MPy, respectively, at 300 K. At 10 K, however, it is 260 μW μm^−2^ for BPT, nearly threefold larger, showing how much harder it is to create picocavities at lower temperature. Similar BPT thresholds are found with 60-nm NPs (fig. S7). Besides this 50-fold variation in critical intensities, the saturation rate *P*_0p_ also depends on molecule type (Fig. 3C, ◇, and table S2). These observations reinforce that optical heating cannot explain the results, since the tripling of creation rate from ambient *T* = 10 to 300 K does not correspond to the 12,000 K required to exceed *U*_f_.

The formation rate of flares (*P*_f_; Fig. 3D) is similarly extracted (note S3) and gives very similar *I*_c_ with *P*_f_ ∝ *P*_p_, indicating that irradiation plays a similar role in both processes. We note that flare creation rates also depend on the molecule species (although the flare spectral shape does not), emphasizing formation from the same light-molecule-metal interaction. All these molecules have the same thiol attachment to the lower Au mirror, suggesting that the molecular-dependent effects here arise from the apex of the molecules.

#### Decay rates

Picocavity decay rates (*R*_p_; Fig. 3, B and F) are extracted from the distribution of lifetimes (*T*_p_ spans 200 ms to 180 s) by performing maximum log-likelihood estimation (fig. S8, note S4, and table S3). A clear double-exponential distribution is seen, showing that picocavities divide into two types (Fig. 3F), fast and slow (~5-fold longer-lived). The longest characteristic lifetimes of 180 s are observed for MPy, despite it has the lowest intensity threshold. Threefold more fast-decaying than slow-decaying picocavities (Fig. 3F, marker size) is perhaps related to their metastable configurations (molecule-metal transient coordination bonds, as discussed below) (*21*).

Previously published molecule-adatom interactions forming these coordination bonds (*32*) resolve the effect of their conformation on single-molecule SERS spectra (figs. S9 and S10 and table S5). The carefully calculated DFT Morse potentials (fig. S11) give free energies of binding $U_{b}^{0}\gg k_{B}T$ (table S6), accounting for their room temperature stability. The slow decay rates are found to be light-activated in a similar way to their creation rates. Decay rates are higher in BPT than in MBN or MPy (again just as for picocavity creation rates), confirming the lower stability of picocavities in BPT as well as the greater difficulty of creating them (Fig. 3E).

Flare decay rates (*R*_f_; Fig. 3G) are similarly calculated (fig. S8 and table S4), again divided into fast and slow types, the latter being >3-fold rarer and ~8-fold longer-lived for BPT, similar to picocavities (few flares for MBN or MPy make fast decay rates hard to quantify). For all molecules, the decay rate of flares is ~10-fold higher than that of picocavities, showing that picocavities are more stable.

## DISCUSSION

Picocavities are not seen initially or at the lowest powers (Fig. 1B), which implies that Au^(0)^ adatoms do not exist on the nanoparticle facets before illumination (which suggests that the strong van der Waals attraction anneals the metal facets completely flat). To understand how light induces Au atom motion, our results point to the influence of molecule type and temperature. The critical intensity for each molecule is found to inversely correlate with their DFT coordination binding energies (Fig. 3E), as well as their decay times. This shows that when a Au adatom can strongly coordinate to the nearby molecule, this aids picocavity creation and retards picocavity decay. The observed exponential waiting time probability *P*_p_(*t*) ∝ exp{−*t*/τ_p_} for a picocavity to form (Fig. 3A) implies a thermally activated process (rather than deterministically driven by the laser). The picocavity creation rate should thus follow

$$P_{p}=P_{0p}\text{exp}\{-U_{f}/k_{B}T\}$$ (1)

As noted, optical heating on its own cannot account for the energies required to abstract Au adatoms, nor is such heating observed in the anti-Stokes ERS, which has been shown to track the metal temperature accurately; if observed temperatures (fig. S12) drive picocavities, this would imply unfeasibly small 5 meV barriers (≪ *U*_f_). Such heating also cannot explain why formation is harder at lower initial temperatures or why it saturates at high laser intensities. In the discussion here, we show that optical forces are far too weak to be directly involved and thus explore alternative explanations.

### Estimate of optical forces (model 1)

**Fig. 4.**
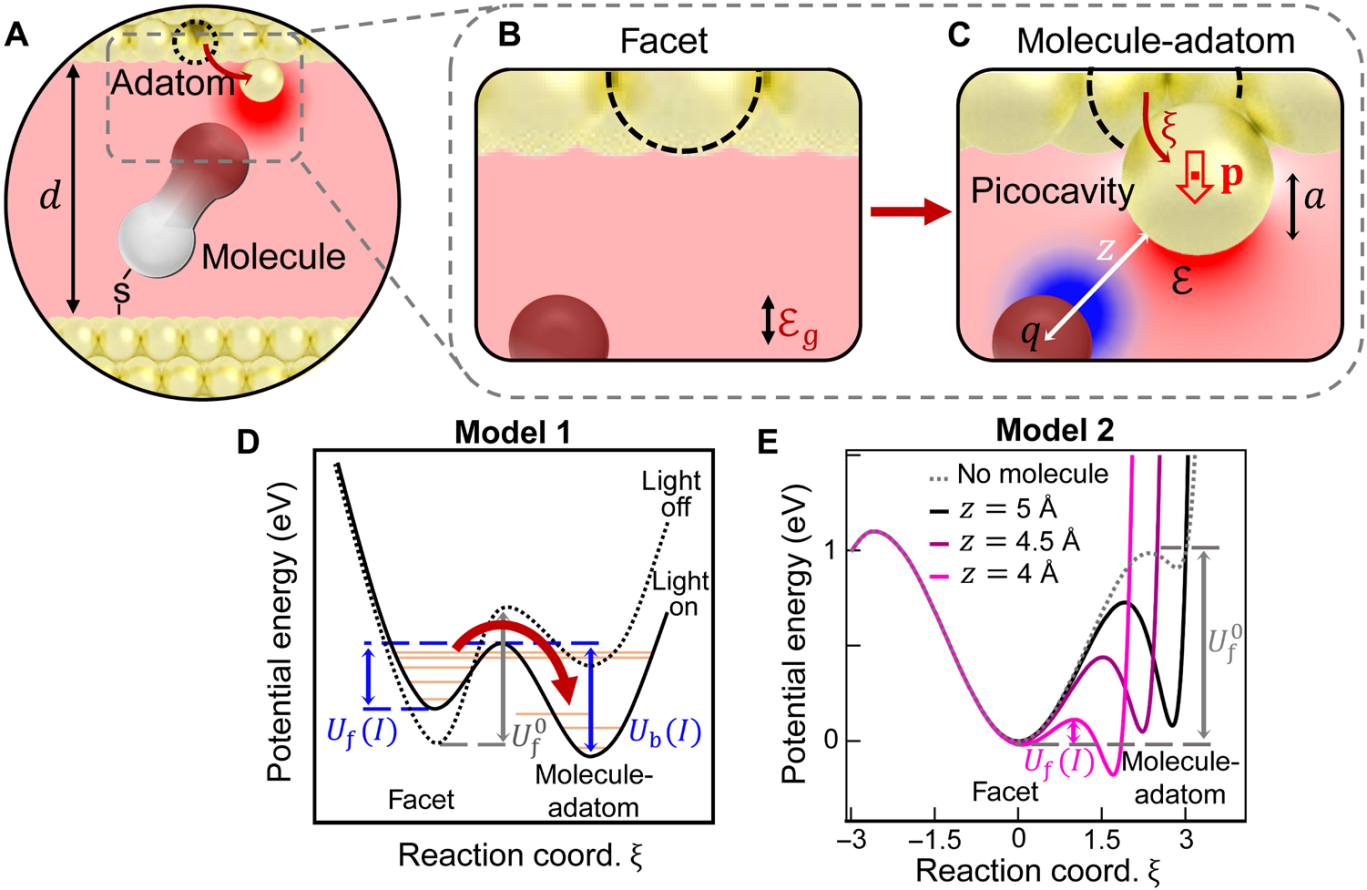
Proposed mechanism for light-driven changes in molecule-metal binding. (**A** to **C**) Scheme of picocavities, with localization of optical field (red) around an adatom, which attracts the molecule tip. Reaction coordinate ξ in (C) is the trajectory distance of the adatom from the initial site in facet of (B), *z* is the distance from the tip atom (radius *a*) to the edge of the Au adatom, and **p** is the dipole induced by gap optical field ℰ_g_ producing picocavity field ℰ. (**D**) Model 1: Optical forces in picocavity field gradient tilt the potential when the laser is on (solid) versus off (dashed). $U_{f}^{0}$ is the barrier for adatom formation when the laser is off, and *U*_f,b_(*I*) are barriers for adatom formation and decay at laser intensity *I*. (**E**) Model 2: Simulated energy for picocavities when molecule tip–adatom separation *z* decreases by light (solid) versus without molecule (dashed), showing reduced barrier height.

To form adatoms, the Au atom moves ~1.5 Å (the atomic radius *a*), thus requiring typical forces $F_{c}=\frac{d}{\mathrm{dr}}U_{f}^{0}\sim U_{f}^{0}/a>\;1$ nN. Confining the incident intensity$\;I=\frac{1}{2}c\epsilon_{0}{|E_{i}|}^{2}$ into the gap (speed of light in vacuum *c*, vacuum permittivity ϵ_0_, and incident light field ℰ_i_) generates optical force $F=\frac{1}{2}\alpha_{a}\nabla {ℰ}^{2}$ from the optical field gradient around the adatom (Figs. 4, A to C, and fig. S14) (*19*), with polarizability α*_a_* = 4πϵ_0_*a*^3^ of the adatom sphere. This yields (notes S6 to S8) an optical force in model 1 of *F*_o_~*I*η ℵ EF^2^*a*^2^/*c*, where η~0.4 is the coupling efficiency into the resonant plasmonic mode (*37*), EF~500 is the local enhancement factor of the optical field in the nanogap center at this resonance (fig. S2), and ℵ~4 accounts for the classical local enhancement (*15*) of the metal protrusion (fig. S15). For the parameters here, *F*_o_~1 pN per mW illumination, which is four to five orders of magnitude too weak to overcome the adatom extraction force *F_c_*. Considering the resulting potential versus Au adatom position ξ (fig. S20), a further problem with model 1 comes from the optical force that tilts the potential (solid line, Fig. 4D). This reduces the forward barrier for adatom creation *U*_f_(*I*), but the barrier for its subsequent relaxation *U*_b_(*I*) is then larger, which implies that adatom decay should not be seen while the light is on. In direct contrast, our observed decay rates match the creation rates (Fig. 3); hence, conventional optical forces cannot be responsible.

Other models considered include hot Au atom excitation (analogous to hot electrons; fig. S23), in which a plasmon gives its entire energy to kick a Au atom over the barrier *U*_f_. However, this should not be temperature or molecule dependent (note S10). We also confirm that quantum tunneling of the Au atom through this barrier has implausibly low probability.

### Light-activated molecule-metal energy barrier reduction (model 2)

For Eq. 1 to match the creation rates observed, the barrier height must reduce with increasing laser intensities. We thus introduce a tentative model to explain how this can occur. We first note that the closest molecule to the adatom influences the locally dressed permittivity in the picocavity field via its effective polarizability α rather than behaving as a uniform dielectric. With the laser on, the adatom acts as an optical dipole [as evidenced in (*15*)], producing field E_t_ at the molecule tip, which polarizes it (Fig. 4C) as p_t_ = α_t_(E_t_ + E_im_) = α_t_ℰ_t_ + α_t_ℵ p/[4πϵ_0_(2*z*)^3^] = αE_t_ where its proximity to the plasmonic metal gives image charges that induce additional field ℰ_im_ at the molecule (fig. S13). Here, α_t_ is the tip atom polarizability and *z* is the separation of the tip atom of the molecule to the classical edge of the adatom. This drastically enhances the effective α (in a quasi-static approximation; see note S8)

$$\frac{\alpha }{\alpha_{t}}\equiv \zeta ≃\frac{1}{1-{\left(\kappa /z\right)}^{3}}$$ (2)

where κ = (α_t_ℵ/8)^1/3^. In a uniform optical field, typical molecular polarizabilities α_0_ ~10 Å^3^ (benzene) give critical separation κ ~1.8 Å, but here, the local polarizability can be much higher since the picocavity field is so nonuniform over a single electronic orbital (Fig. 4C), as estimated by DFT below. In this semiclassical model, for *z* < κ, the polarizability becomes infinite, a “polarization catastrophe” thought sufficient to deform/break molecules when close to metals (*38*). The resulting dipole-dipole (or induced van der Waals) attraction between the picocavity adatom dipole and the induced molecular dipole enhances the force *F*_p_~*F*_o_ α/α_0_ by >10^3^. The local polarizability at the molecule tip creates opposite charges at the adatom, inducing an attractive optical force (model 2; Fig. 4E). The attracted molecule comes even closer to the nanoparticle facet, reducing the energy barrier for adatom protrusion. The binding enhances as the molecule approaches, until quantum–wave function repulsion impedes it enough to set the equilibrium molecular separation from the facet.

**Fig. 5.**
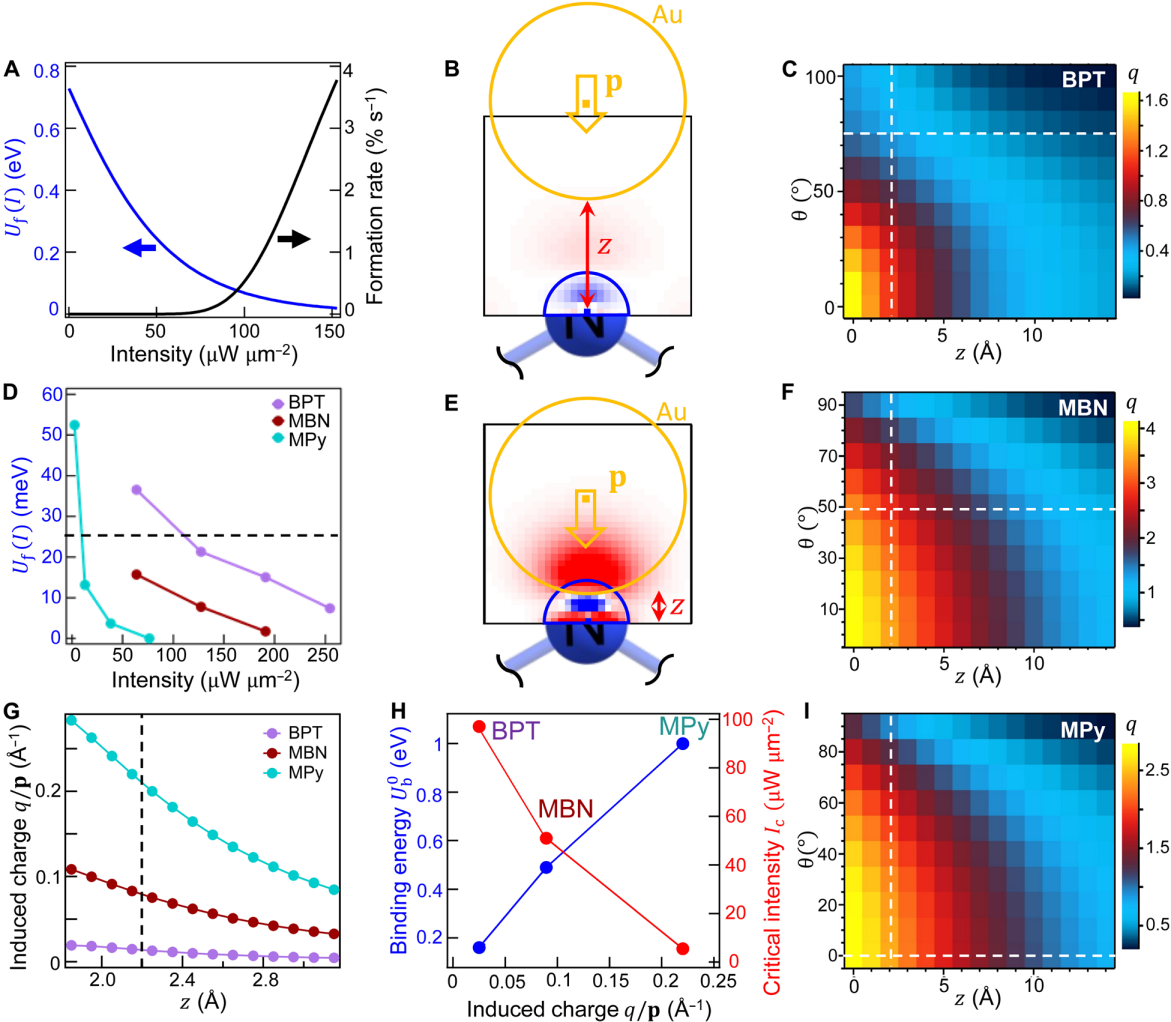
Light-induced molecule tip atom local polarizability relation to intensity threshold. (**A**) Simulated dependence of *U*_f_(*I*) and picocavity formation rate versus laser power in model 2 for BPT. (**B** and **E**) DFT-calculated charge *q* induced on the tip atom (here, N for MPy) when the Au adatom field is treated as an optical dipole of strength **p** at distance *z* away. (**C**, **F**, and **I**) Induced charge *q*, mapped versus adatom position, with *z* and angle θ on planes perpendicular to the aromatic ring (see fig. S10). Dashed lines show energetically favorable adatom from DFT at *z* = 2.2 Å and θ = 75°, 49°, and 0° for the three molecules. (**D**) Extracted *U*_f_(*I*) for different molecules from data, thresholds given by *U*_f_(*I*_c_) = *k*_B_*T* (dashed). (**G**) Induced charge *q* from DFT tracks tip atom polarizability α_t_. (**H**) Molecule-metal binding energy well depth $U_{b}^{0}$ and critical laser intensity *I*_c_ required for picocavity and flare formation versus induced charge *q* at the molecule tip atom.

Above the critical intensity, the barrier becomes low enough for thermal excitation to occur, giving observable picocavities. A simple simulation using this model (fig. S22) indeed suggests that the net energy barrier is controlled by light intensity (Fig. 5A). From Eq. 1, we extract *U*_f_(*I*), which decreases with power in experiment (Fig. 5D), giving critical intensity at *U*_f_(*I*_c_) = *k*_B_*T* (dashed line). In this model, both creation and decay of picocavities are similarly light-activated by the barrier reduction (as observed), in contrast to all other models considered. The saturation rate *P*_0p_ (Fig. 3C) is then controlled by the minimum tip atom–to–adatom separation that is quantum mechanically feasible. Irradiation decreases the barrier height as E^−2^, so adatoms are pulled out only in the strongest near-field positions E_g_ within the nanogap, dependent on the precise alignment of the closest molecule to prospective adatoms in the facet, as shown in recent experiments (*33*).

The influence of different molecules at the metal surface can now be related to the tip atom polarizability from local dipolar excitation, which thus decreases barrier height faster for MPy than for BPT. DFT modeling is performed on each molecule (see Materials and Methods), with the tip atom being C for BPT (approaching from out of plane) and N for MBN and MPy. The alignment of the Au adatom is first determined from the minimum energy conformation with relative position (figs. S10 and S11 and table S5). The optical excitation is then treated as a dipole of strength p at the Au adatom (Fig. 5, B and E). As the dipole approaches the molecular tip, the induced attractive optical force pulls electrons toward the frontier orbital of the molecular tip atom, changing the charge distribution around it (fig. S17). The resulting induced charge *q* at the molecular tip atom is mapped versus the relative position of the Au adatom (Fig. 5, C, F, and I). To extract α_t_, *q* is then multiplied by cos^2^θ (accounting for the orientation of optical field and projection of vector polarizability; note S8) to generate the component of polarizability in the vertical direction (*q*/p; Fig. 5G). At theoretical equilibrium separations of *z* ~ 2.2 Å (dashed, similar for all these molecules in DFT; also see fig. S11), the induced charge is ~20-fold larger for MPy than for BPT. This is consistent with the ratio of threshold intensities for picocavity formation, as *I*_c_ is ~20-fold smaller for MPy than for BPT (Fig. 5H). All the light-induced barriers *U*_f_(α_t_*I*) collapse onto a universal curve (fig. S21), well fit by $U_{f}(I)=U_{f}^{0}/(I/I_{t}+1)$ (giving Fig. 3C, solid lines), where $I_{c}=I_{t}U_{f}^{0}/k_{B}T$.

Optical effects at molecular-metal interfaces are thus drastically different from uniform dielectrics. To treat them fully consistently, we await quantum mechanical solutions for the local optical polarizability at molecules and atomistically modeled metals including treatment of image charges in continuum electromagnetics. However, here we outlined and evidenced the concept of local polarizability at the molecule tip, which controls the energetic landscape (note S9). While the dynamic behavior of metal-molecule nanojunctions previously implicated molecular properties (*39*, *40*), here we demonstrate that it is molecular coordination with adatoms that is important. We also note that our model explains why picocavity decay rates match creation rates, since the light similarly reduces the barrier for adatoms dropping back into the facet. The similar dependence of rates for picocavities and flares also suggests that in flares, many atoms are polarized in parallel by the light.

Our suggested “optical plucking” (model 2) also leaves issues to be resolved. Previous experiments (*21*, *30*) imply that picocavities can form at the bottom gold mirror. The same mechanism is thus likely at work, inducing polarizabilities of the Au-thiol bond but has higher *I*_c_. Although thiols themselves can pull out single gold atoms from the surface, such “staples” (*41*) are present as Au^(I)^, which is not metallic and does not give picocavity field enhancement. While additional lattice strain might form Au^(0)^, picocavities are not seen before prolonged exposure to light (Fig. 1B), and the thiol would not be affected by the molecular tip (since S-Au binding is little affected by molecule type). Another issue is the possible relaxation of the remaining atoms surrounding the pit when the adatom is pulled out, leading to metastable configurations. Steric considerations must also be important, since the Au adatom is evidently able to move into the space within the dense SAM.

These experiments demonstrate that light-driven attraction between metal and molecules can promote adatoms and stabilize them for many minutes under ambient conditions. Examining possible mechanisms identifies one that accounts for all observations and is supported by DFT calculations of the local polarizability at the molecule tip closest to the Au. Our findings are not limited to optical irradiation, since applying voltage or injection of electron beams (*31*) can also induce adatom formation, offering scope for further study. We also observe that adatom formation rates vary on different facets (note S9), opening up complementary future directions to systematically resolve the effects of facet surface structure on picocavity formation, which are of interest in supramolecular chemistry and nanoparticle synthesis.

Our observations apply to many systems, from optically irradiated molecular electronics and photocatalysis to semiconductor-metal optoelectronic devices. In all cases, interactions between a polarizable atom and a metallic atom can create extremely powerful optical forces capable of rearranging the material interface. Detailed observations here come from using precise plasmonic constructs that efficiently and reproducibly trap light as well as providing the sensitivity to quantify what structural changes are occurring. While such plasmonic nanogaps are ideal model systems, similar optical confinement is found in many granular metal photocatalysts or at the sharp asperities of individual nanoparticles, implying that optical restructuring of surfaces is widespread. The model that emerges provides intuition for using light-molecule-metal systems to control single-atom optical switches, photocatalysis, photovoltaics, and sensing. However, this work should also provide strong encouragement to develop new theories capable of combining electromagnetism with quantum mechanics.

## MATERIALS AND METHODS

### Experimental design

#### Sample preparation

All chemicals were purchased from Sigma-Aldrich and used as received, unless stated otherwise. Atomically smooth gold substrates were fabricated by template-stripping methods (*19*, *21*). SAMs of BPT, MBN (Santa Cruz Biotechnology), and MPy were formed by immersing the substrates in anhydrous ethanol solution, at concentrations of 1, 2, and 2 mM, respectively, for 16 hours. The substrates were rinsed with ethanol and dried with nitrogen. Standard AuNPs in citrate buffer were purchased from BBI Solutions (*42*) with reported morphology (*43*). AuNPs were deposited by drop-casting either 80 nm AuNP solution onto BPT samples for 60 s, or 60 nm AuNP solution mixed with 40 mM NaNO_3_ onto MBN and MPy samples for 20 s. These AuNP sizes are chosen because of their stronger optical response (∝*D*^6^) and high stability. Measuring anti-Stokes–to–Stokes ratios proves that optical heating in our system is below 10 K here so the gold remains at near room temperature (*T* < 50°C) and no melting occurs (melting point for these NPs, ~1000°C), as plasmons do not directly heat the molecules (*19*) and induced optical heating is negligible (*44*). The samples were rinsed with deionized water and dried. No SERS signatures of citrate are ever seen from the NPoMs, as expected from the larger binding affinity of thiols that displace them.

#### SERS measurements

All spectra were recorded on a modified Olympus BX51 microscope coupled to a Raman spectrometer. A Prior Scientific motorized stage was used to move the sample. Dark-field images were recorded using a Lumenera Infinity2 charge-coupled device (CCD) camera. A 632.8-nm single-frequency diode laser was used as the excitation source. Intensities were derived from the laser power on the sample measured using a Thorlabs PM16-121 power meter. For room temperature measurements, the laser was focused on a diffraction-limited spot of diameter ~1 μm. Integration times used for BPT, MBN, and MPy were 0.2, 1, and 1 s, respectively. Excitation and collection were performed using a dichroic beamsplitter and an Olympus MPLFLN100XBD NA (numerical aperture) 0.9 objective. Elastically scattered laser light was removed using two Thorlabs NF633-25 notch filters. Scattered light was imaged onto an Andor Newton EMCCD coupled to a HORIBA Triax 320 spectrometer. For low-temperature measurements, the laser was focused on a ~2-μm-diameter spot, and an integration time of 3 s was used. An Oxford Instruments Microstat HiRes cryostat, an Olympus LMPLFLN100XBD NA 0.8 objective, and two Semrock NF03-633E-25 single-notch filters were used. Scattered light was imaged on an Andor Newton EMCCD coupled to an Andor Shamrock 303i spectrometer.

### DFT calculations

Molecules were modeled with thiol groups bound to single gold atoms for nanocavity systems (Fig. 2) and to two gold atoms for picocavity systems (fig. S3) to ensure an even number of electrons. Adatoms were modeled as single gold atoms attached to the molecules. Compared with DFT of molecules bound to large gold layers, DFT from binding to one or two gold atoms matches the experimental SERS well (*21*, *45*) at much lower computational cost. Gas-phase geometry optimizations, frequency calculations, and calculations for changes in electron density (Fig. 5) were carried out with no symmetry restrictions. Grimme’s D3 dispersion correction with Becke-Johnson damping GD3BJ (*46*) was used with the B3LYP hybrid functional and the def2TZVP basis set. The ultrafine integration grid was used to enhance the accuracy of calculations. To match the experiment, computational spectra were scaled by a factor of 0.97. The external electric field was modeled in the computations via two +0.1 e and −0.1 e point charges, forming a dipole at grid points near the N atoms (MPy and MBN) or the C atom (BPT) of the molecule. The distance between the point charges is 0.1 Å in all calculations, and the dipole vector points toward the N or C atoms. Changes in electron density were calculated with respect to the unperturbed system. All DFT calculations were carried out with the Gaussian09 Revision E (*47*).

### Statistical analysis

Sample sizes (*n*) are indicated in the captions of Fig. 3 and fig. S7. Formation rates (*P*, in second^−1^) are calculated from the fraction of empty time series where picocavities or flares are not observed (*P*_empty_), with details in note S3. Decay rates were extracted by modeling the lifetime (*T*, in seconds) of each transient event with a biexponential probability density function, with fractions of fast and slow events as detailed in note S4. The statistical results are summarized in tables S1 to S3 and plotted in Fig. 3.

## Supplementary Materials

### This PDF file includes:

Notes S1 to S10 Figs. S1 to S23 Tables S1 to S6 References

## References

[1] B. S. Hoener, S. R. Kirchner, T. S. Heiderscheit, S. S. E. Collins, W. S. Chang, S. Link, C. F. Landes, Plasmonic sensing and control of single-nanoparticle electrochemistry. Chem 4, 1560–1585 (2018).

[2] L. Zhou, D. F. Swearer, C. Zhang, H. Robatjazi, H. Zhao, L. Henderson, L. Dong, P. Christopher, E. A. Carter, P. Nordlander, N. J. Halas, Quantifying hot carrier and thermal contributions in plasmonic photocatalysis. Science 362, 69–72 (2018). 30287657 10.1126/science.aat6967

[3] G. Li, R. Zhu, Y. Yang, Polymer solar cells. Nat. Photonics 6, 153–161 (2012).

[4] B. Sepúlveda, P. C. Angelomé, L. M. Lechuga, L. M. Liz-Marzán, LSPR-based nanobiosensors. Nano Today 4, 244–251 (2009).

[5] P. K. Jain, X. Huang, I. H. El-Sayed, M. A. El-Sayed, Noble metals on the nanoscale: Optical and photothermal properties and some applications in imaging, sensing, biology, and medicine. Acc. Chem. Res. 41, 1578–1586 (2008). 18447366 10.1021/ar7002804

[6] M. Galperin, Photonics and spectroscopy in nanojunctions: A theoretical insight. Chem. Soc. Rev. 46, 4000–4019 (2017). 28398449 10.1039/c7cs00067g

[7] N. Xin, J. Guan, C. Zhou, X. Chen, C. Gu, Y. Li, M. A. Ratner, A. Nitzan, J. F. Stoddart, X. Guo, Concepts in the design and engineering of single-molecule electronic devices. Nat. Rev. Phys. 1, 211–230 (2019).

[8] P. Gehring, J. M. Thijssen, H. S. J. van der Zant, Single-molecule quantum-transport phenomena in break junctions. Nat. Rev. Phys. 1, 381–396 (2019).

[9] A. R. Rocha, V. M. García-suárez, S. W. Bailey, C. J. Lambert, J. Ferrer, S. Sanvito, Towards molecular spintronics. Nat. Mater. 4, 335–339 (2005). 15750597 10.1038/nmat1349

[10] L. Bogani, W. Wernsdorfer, Molecular spintronics using single-molecule magnets. Nat. Mater. 7, 179–186 (2008). 18297126 10.1038/nmat2133

[11] S. Sanvito, Molecular spintronics. Chem. Soc. Rev. 40, 3336–3355 (2011). 21552606 10.1039/c1cs15047b

[12] S. Schlücker, Surface-enhanced raman spectroscopy: Concepts and chemical applications. Angew. Chemie Int. Ed. 53, 4756–4795 (2014). 10.1002/anie.201205748 24711218

[13] M. Barbry, P. Koval, F. Marchesin, R. Esteban, A. G. Borisov, J. Aizpurua, D. Sánchez-Portal, Atomistic near-field nanoplasmonics: Reaching atomic-scale resolution in nanooptics. Nano Lett. 15, 3410–3419 (2015). 25915173 10.1021/acs.nanolett.5b00759

[14] S. Trautmann, J. Aizpurua, I. Götz, A. Undisz, J. Dellith, H. Schneidewind, M. Rettenmayr, V. Deckert, A classical description of subnanometer resolution by atomic features in metallic structures. Nanoscale 9, 391–401 (2017). 27924333 10.1039/c6nr07560f

[15] M. Urbieta, M. Barbry, Y. Zhang, P. Koval, D. Sánchez-Portal, N. Zabala, J. Aizpurua, Atomic-scale lightning rod effect in plasmonic picocavities: A classical view to a quantum effect. ACS Nano 12, 585–595 (2018). 29298379 10.1021/acsnano.7b07401

[16] R. Zhang, Y. Zhang, Z. C. Dong, S. Jiang, C. Zhang, L. G. Chen, L. Zhang, Y. Liao, J. Aizpurua, Y. Luo, J. L. Yang, J. G. Hou, Chemical mapping of a single molecule by plasmon-enhanced Raman scattering. Nature 498, 82–86 (2013). 23739426 10.1038/nature12151

[17] J. Lee, K. T. Crampton, N. Tallarida, V. A. Apkarian, Visualizing vibrational normal modes of a single molecule with atomically confined light. Nature 568, 78–82 (2019). 30944493 10.1038/s41586-019-1059-9

[18] M. Richard-Lacroix, V. Deckert, Direct molecular-level near-field plasmon and temperature assessment in a single plasmonic hotspot. Light Sci. Appl. 9, 35 (2020). 32194949 10.1038/s41377-020-0260-9 PMC7061098

[19] F. Benz, M. K. Schmidt, A. Dreismann, R. Chikkaraddy, Y. Zhang, A. Demetriadou, C. Carnegie, H. Ohadi, B. de Nijs, R. Esteban, J. Aizpurua, J. J. Baumberg, Single-molecule optomechanics in “picocavities”. Science 354, 726–729 (2016). 27846600 10.1126/science.aah5243

[20] H.-H. Shin, G. J. Yeon, H.-K. Choi, S.-M. Park, K. S. Lee, Z. H. Kim, Frequency-domain proof of the existence of atomic-scale SERS hot-spots. Nano Lett. 18, 262–271 (2018). 29206468 10.1021/acs.nanolett.7b04052

[21] C. Carnegie, J. Griffiths, B. de Nijs, C. Readman, R. Chikkaraddy, W. M. Deacon, Y. Zhang, I. Szabó, E. Rosta, J. Aizpurua, J. J. Baumberg, Room-temperature optical picocavities below 1 nm^3^ accessing single-atom geometries. J. Phys. Chem. Lett. 9, 7146–7151 (2018). 30525662 10.1021/acs.jpclett.8b03466

[22] J. Huang, D.-B. Grys, J. Griffiths, B. de Nijs, M. Kamp, Q. Lin, J. J. Baumberg, Tracking interfacial single-molecule pH and binding dynamics via vibrational spectroscopy. Sci. Adv. 7, eabg1790 (2021). 34088670 10.1126/sciadv.abg1790 PMC8177700

[23] L. Vitos, A. V. Ruban, H. L. Skriver, J. Kollár, The surface energy of metals. Surf. Sci. 411, 186–202 (1998).

[24] J. Takano, O. Takai, Y. Kogure, M. Doyama, Simulation of atomic-scale surface migration in homoepitaxial growth using embedded-atom method potentials for gold. Thin Solid Films 318, 52–56 (1998).

[25] Y. Liu, V. Ozolins, Self-assembled monolayers on Au(111): Structure, energetics, and mechanism of reconstruction lifting. J. Phys. Chem. C 116, 4738–4747 (2012).

[26] D. Thompson, J. Liao, M. Nolan, A. J. Quinn, C. A. Nijhuis, C. O’Dwyer, P. N. Nirmalraj, C. Schönenberger, M. Calame, Formation mechanism of metal–molecule–metal junctions: Molecule-assisted migration on metal defects. J. Phys. Chem. C 119, 19438–19451 (2015).

[27] T.-S. Lin, Y.-W. Chung, Measurement of the activation energy for surface diffusion in gold by scanning tunneling microscopy. Surf. Sci. 207, 539–546 (1989).

[28] J. J. Baumberg, J. Aizpurua, M. H. Mikkelsen, D. R. Smith, Extreme nanophotonics from ultrathin metallic gaps. Nat. Mater. 18, 668–678 (2019). 30936482 10.1038/s41563-019-0290-y

[29] J. Langer, D. J. de Aberasturi, J. Aizpurua, R. A. Alvarez-Puebla, B. Auguié, J. J. Baumberg, G. C. Bazan, S. E. J. Bell, A. Boisen, A. G. Brolo, J. Choo, D. Cialla-May, V. Deckert, L. Fabris, K. Faulds, F. J. G. de Abajo, R. Goodacre, D. Graham, A. J. Haes, C. L. Haynes, C. Huck, T. Itoh, M. Käll, J. Kneipp, N. A. Kotov, H. Kuang, E. C. Le Ru, H. K. Lee, J.-F. Li, X. Y. Ling, S. A. Maier, T. Mayerhöfer, M. Moskovits, K. Murakoshi, J.-M. Nam, S. Nie, Y. Ozaki, I. Pastoriza-Santos, J. Perez-Juste, J. Popp, A. Pucci, S. Reich, B. Ren, G. C. Schatz, T. Shegai, S. Schlücker, L.-L. Tay, K. G. Thomas, Z.-Q. Tian, R. P. Van Duyne, T. Vo-Dinh, Y. Wang, K. A. Willets, C. Xu, H. Xu, Y. Xu, Y. S. Yamamoto, B. Zhao, L. M. Liz-Marzán, Present and future of surface-enhanced raman scattering. ACS Nano 14, 28–117 (2020). 31478375 10.1021/acsnano.9b04224 PMC6990571

[30] C. Carnegie, M. Urbieta, R. Chikkaraddy, B. de Nijs, J. Griffiths, W. M. Deacon, M. Kamp, N. Zabala, J. Aizpurua, J. J. Baumberg, Flickering nanometre-scale disorder in a crystal lattice tracked by plasmonic flare light emission. Nat. Commun. 11, 682 (2020). 32015332 10.1038/s41467-019-14150-w PMC6997371

[31] F. Benz, B. de Nijs, C. Tserkezis, R. Chikkaraddy, D. O. Sigle, L. Pukenas, S. D. Evans, J. Aizpurua, J. J. Baumberg, Generalized circuit model for coupled plasmonic systems. Opt. Express 23, 33255–33269 (2015). 26831992 10.1364/OE.23.033255

[32] J. Griffiths, T. Földes, B. de Nijs, R. Chikkaraddy, D. Wright, W. M. Deacon, D. Berta, C. Readman, D. B. Grys, E. Rosta, J. J. Baumberg, Resolving sub-angstrom ambient motion through reconstruction from vibrational spectra. Nat. Commun. 12, 6759 (2021). 34799553 10.1038/s41467-021-26898-1 PMC8604935

[33] J. Griffiths, B. de Nijs, R. Chikkaraddy, J. J. Baumberg, Locating single-atom optical picocavities using wavelength-multiplexed raman scattering. ACS Photonics 8, 2868–2875 (2021). 34692898 10.1021/acsphotonics.1c01100 PMC8532146

[34] Y.-Y. Cai, E. Sung, R. Zhang, L. J. Tauzin, J. G. Liu, B. Ostovar, Y. Zhang, W.-S. Chang, P. Nordlander, S. Link, Anti-stokes emission from hot carriers in gold nanorods. Nano Lett. 19, 1067–1073 (2019). 30657694 10.1021/acs.nanolett.8b04359

[35] B. de Nijs, R. W. Bowman, L. O. Herrmann, F. Benz, S. J. Barrow, J. Mertens, D. O. Sigle, R. Chikkaraddy, A. Eiden, A. Ferrari, O. A. Scherman, J. J. Baumberg, Unfolding the contents of sub-nm plasmonic gaps using normalising plasmon resonance spectroscopy. Faraday Discuss. 178, 185–193 (2015). 25793557 10.1039/c4fd00195h

[36] J. N. Sweet, The spectral similarity scale and its application to the classification of hyperspectral remote sensing data, in *IEEE Workshop on Advances in Techniques for Analysis of Remotely Sensed Data* (IEEE, 2003), pp. 92–99.

[37] N. Kongsuwan, A. Demetriadou, M. Horton, R. Chikkaraddy, J. J. Baumberg, O. Hess, Plasmonic nanocavity modes: From near-field to far-field radiation. ACS Photonics 7, 463–471 (2020).

[38] C. M. Teodorescu, Image molecular dipoles in surface enhanced Raman scattering. Phys. Chem. Chem. Phys. 17, 21302–21314 (2015). 25669606 10.1039/c4cp05082g

[39] W. Du, T. Wang, H.-S. Chu, L. Wu, R. Liu, S. Sun, W. K. Phua, L. Wang, N. Tomczak, C. A. Nijhuis, On-chip molecular electronic plasmon sources based on self-assembled monolayer tunnel junctions. Nat. Photonics 10, 274–280 (2016).

[40] T. Wang, W. Du, N. Tomczak, L. Wang, C. A. Nijhuis, In operando characterization and control over intermittent light emission from molecular tunnel junctions via molecular backbone rigidity. Adv. Sci. 6, 1900390 (2019). 10.1002/advs.201900390 PMC6794720 31637155

[41] H. Häkkinen, The gold–sulfur interface at the nanoscale. Nat. Chem. 4, 443–455 (2012). 22614378 10.1038/nchem.1352

[42] BBI Solutions, *Diagnostic Gold Colloid*; www.bbisolutions.com/en/reagents/gold-colloid.

[43] F. Benz, R. Chikkaraddy, A. Salmon, H. Ohadi, B. de Nijs, J. Mertens, C. Carnegie, R. W. Bowman, J. J. Baumberg, SERS of individual nanoparticles on a mirror: Size does matter, but so does shape. J. Phys. Chem. Lett. 7, 2264–2269 (2016). 27223478 10.1021/acs.jpclett.6b00986 PMC4916483

[44] G. Baffou, R. Quidant, F. J. García de Abajo, Nanoscale control of optical heating in complex plasmonic systems. ACS Nano 4, 709–716 (2010). 20055439 10.1021/nn901144d

[45] D. Wright, Q. Lin, D. Berta, T. Földes, A. Wagner, J. Griffiths, C. Readman, E. Rosta, E. Reisner, J. J. Baumberg, Mechanistic study of an immobilized molecular electrocatalyst by in situ gap-plasmon-assisted spectro-electrochemistry. Nat. Catal. 4, 157–163 (2021).

[46] A. Allouche, Software news and updates Gabedit—A graphical user interface for computational chemistry softwares. J. Comput. Chem. 32, 174–182 (2012). 10.1002/jcc.21600 20607691

[47] M. J. Frisch, G. W. Trucks, H. B. Schlegel, G. E. Scuseria, M. A. Robb, J. R. Cheeseman, G. Scalmani, V. Barone, B. Mennucci, G. A. Petersson, H. Nakatsuji, M. Caricato, X. Li, H. P. Hratchian, A. F. Izmaylov, J. Bloino, G. Zheng, J. L. Sonnenberg, M. Hada, M. Ehara, K. Toyota, R. Fukuda, J. Hasegawa, M. Ishida, T. Nakajima, Y. Honda, O. Kitao, H. Nakai, T. Vreven, J. A. Montgomery, J. E. Peralta, F. Ogliaro, M. Bearpark, J. J. Heyd, E. Brothers, K. N. Kudin, V. N. Staroverov, R. Kobayashi, J. Normand, K. Raghavachari, A. Rendell, J. C. Burant, S. S. Iyengar, J. Tomasi, M. Cossi, N. Rega, J. M. Millam, M. Klene, J. E. Knox, J. B. Cross, V. Bakken, C. Adamo, J. Jaramillo, R. Gomperts, R. E. Stratmann, O. Yazyev, A. J. Austin, R. Cammi, C. Pomelli, J. W. Ochterski, R. L. Martin, K. Morokuma, V. G. Zakrzewski, G. A. Voth, P. Salvador, J. J. Dannenberg, S. Dapprich, A. D. Daniels, Ö. Farkas, J. B. Foresman, J. V Ortiz, J. Cioslowski, D. J. Fox, Gaussian 09 Revision E (2009).

[48] T. Giannakopoulos, PyAudioAnalysis: An open-source python library for audio signal analysis. PLOS ONE 10, e0144610 (2015). 26656189 10.1371/journal.pone.0144610 PMC4676707

[49] A. Moroz, Depolarization field of spheroidal particles. J. Opt. Soc. Am. B. 26, 517 (2009).

[50] E. M. Purcell, D. J. Morin, *Electricity and Magnetism* (Cambridge Univ. Press, ed. 3, 2013).

[51] F. D. Nunes, T. C. Vasconcelos, M. Bezerra, J. Weiner, Electromagnetic energy density in dispersive and dissipative media. J. Opt. Soc. Am. B. 28, 1544–1552 (2011).

[52] A. Ashkin, Optical trapping and manipulation of neutral particles using lasers. Proc. Natl. Acad. Sci. 94, 4853–4860 (1997). 9144154 10.1073/pnas.94.10.4853 PMC24595

[53] D.-B. Grys, B. de Nijs, A. R. Salmon, J. Huang, W. Wang, W.-H. Chen, O. A. Scherman, J. J. Baumberg, Citrate coordination and bridging of gold nanoparticles: The role of gold adatoms in AuNP aging. ACS Nano 14, 8689–8696 (2020). 32543831 10.1021/acsnano.0c03050 PMC7467807

[54] M. Giesen, Step and island dynamics at solid/vacuum and solid/liquid interfaces. Prog. Surf. Sci. 68, 1–154 (2001).

[55] N. V. Smith, C. T. Chen, M. Weinert, Distance of the image plane from metal surfaces. Phys. Rev. B. 40, 7565–7573 (1989). 10.1103/physrevb.40.7565 9991183

[56] A. Xomalis, R. Chikkaraddy, E. Oksenberg, I. Shlesinger, J. Huang, E. C. Garnett, A. F. Koenderink, J. J. Baumberg, Controlling optically driven atomic migration using crystal-facet control in plasmonic nanocavities. ACS Nano 14, 10562–10568 (2020). 32687323 10.1021/acsnano.0c04600 PMC7458481

[57] M. J. Horton, O. S. Ojambati, R. Chikkaraddy, W. M. Deacon, N. Kongsuwan, A. Demetriadou, O. Hess, J. J. Baumberg, Nanoscopy through a plasmonic nanolens. Proc. Natl. Acad. Sci. 117, 2275–2281 (2020). 31941710 10.1073/pnas.1914713117 PMC7006646

[58] M. Grzelczak, J. Pérez-Juste, P. Mulvaney, L. M. Liz-Marzán, Shape control in gold nanoparticle synthesis. Chem. Soc. Rev. 37, 1783–1791 (2008). 18762828 10.1039/b711490g

[59] O. S. Ojambati, R. Chikkaraddy, W. M. Deacon, J. Huang, D. Wright, J. J. Baumberg, Efficient generation of two-photon excited phosphorescence from molecules in plasmonic nanocavities. Nano Lett. 20, 4653–4658 (2020). 32422048 10.1021/acs.nanolett.0c01593 PMC7366501

[60] C. Latouche, Y.-R. Lin, Y. Tobon, E. Furet, J.-Y. Saillard, C.-W. Liu, A. Boucekkine, Au–Au chemical bonding induced by UV irradiation of dinuclear gold(I) complexes: A computational study with experimental evidence. Phys. Chem. Chem. Phys. 16, 25840–25845 (2014). 25352132 10.1039/c4cp03990d

[61] H. Kuramochi, S. Takeuchi, M. Iwamura, K. Nozaki, T. Tahara, Tracking photoinduced Au–Au bond formation through transient terahertz vibrations observed by femtosecond time-domain raman spectroscopy. J. Am. Chem. Soc. 141, 19296–19303 (2019). 31774668 10.1021/jacs.9b06950

