# Supplementary material for: Optical suppression of energy barriers in single molecule-metal binding
Source: Sci Adv. 2022 Jun 24;8(25):eabp9285. doi: 10.1126/sciadv.abp9285 (PMC9232110; doi:10.1126/sciadv.abp9285)
Supplement: Supplementary file 1 — Notes S1 to S10 Figs. S1 to S23 Tables S1 to S6 References [file sciadv.abp9285_sm.pdf]

Science Advances
AAAS

Supplementary Materials for

Optical suppression of energy barriers in single molecule-metal binding

Qianqi Lin *et al.*

Corresponding author: Jeremy J. Baumberg, jjb12@cam.ac.uk

Sci. Adv. **8**, eabp9285 (2022)
DOI: 10.1126/sciadv.abp9285

This PDF file includes:

Notes S1 to S10
Figs. S1 to S23
Tables S1 to S6
References

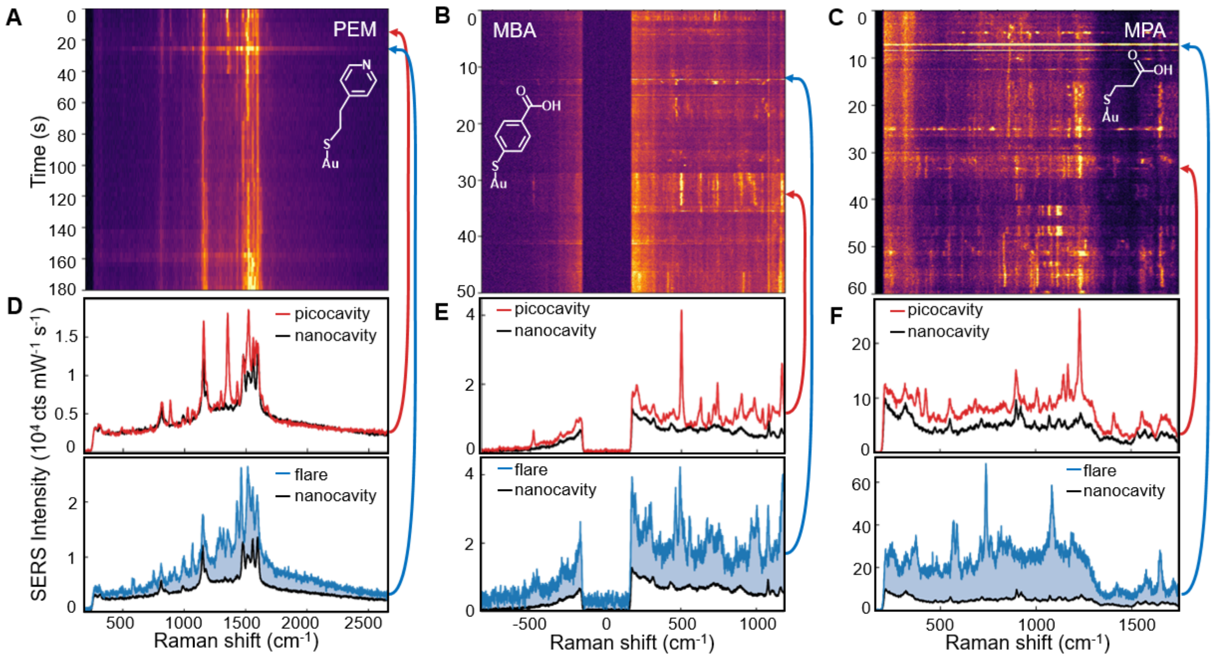

Fig. S1. Further Raman spectra showing evolution of adatoms and adlayers. (**A**) Time-series SERS spectra of 4-4-pyridylethylmercaptan (PEM) for 20 µW 633 nm laser irradiation, exposure time 3s per spectrum. (**B**) Time-series SERS spectra of 4-mercaptobenzoic acid (MBA) for 580 µW irradiation, exposure times 0.1s. (**C**) Time-series SERS spectra of 3-mercaptopropionic acid (MPA) for 1 mW irradiation, exposure times 0.1s. (**D-F**) Example SERS spectra from the nanocavity (black, averaged), a picocavity (red), and a flare (blue). Insets of molecular structures shown in (**A-B**).

Supplementary Note S1. Dark-field scattering spectroscopy

Dark-field spectroscopy was performed on a modified Olympus BX51 coupled to an Ocean Optics QE65000 spectrometer using a 50 µm optical fibre. An incoherent white light source was used as excitation source. Excitation and collection were through an Olympus LMPLFLN100XBD NA 0.8 objective. Automated scans were run to measure many hundreds of individual NPoMs across the sample, using a Python particle tracking code.(*35*) A standard diffuser was used as a reference to normalise the scattering spectra.

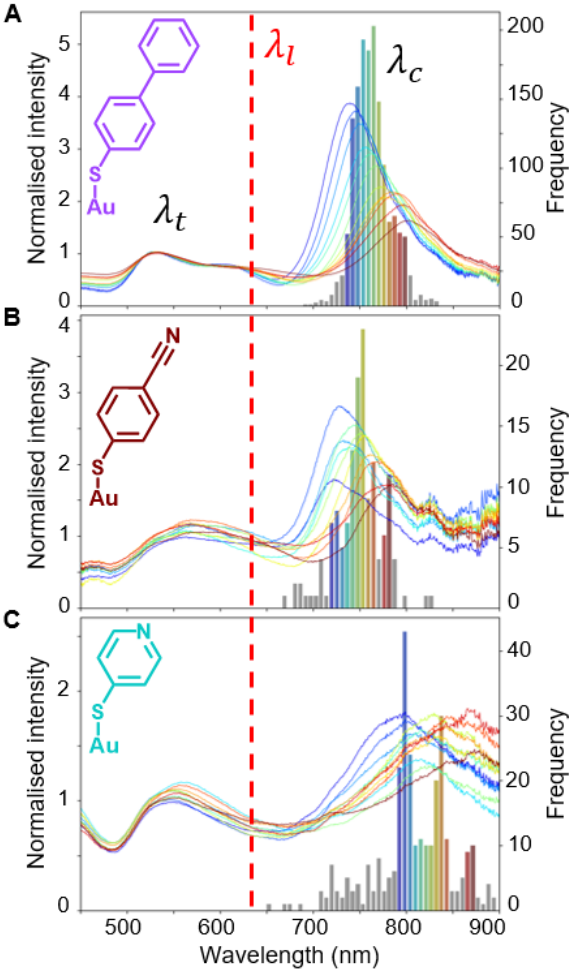

Fig. S2. Dark-field scattering spectroscopy. Spectra of molecular monolayers in NPoMs with overlaid histogram of the coupled mode (λ_c) positions. λ_t is the transverse mode and λ_l = 633 nm is the laser wavelength used for SERS. (**A**) BPT from 1570 NPoMs with 80 nm diameter, $\lambda_c \sim 760$ nm. (**B**) MBN from 548 NPoMs with 60 nm diameter, $\lambda_c \sim 752$ nm. **C,** MPy from 503 NPoMs with 60 nm diameter, $\lambda_c \sim 812$ nm. Different nanoparticle sizes were chosen to optimise λ_c for near-resonant irradiation. NPoMs with diameter less than 60 nm blue-shift λ_c closer to λ_l, but scatter too weakly to be observed in dark-field.

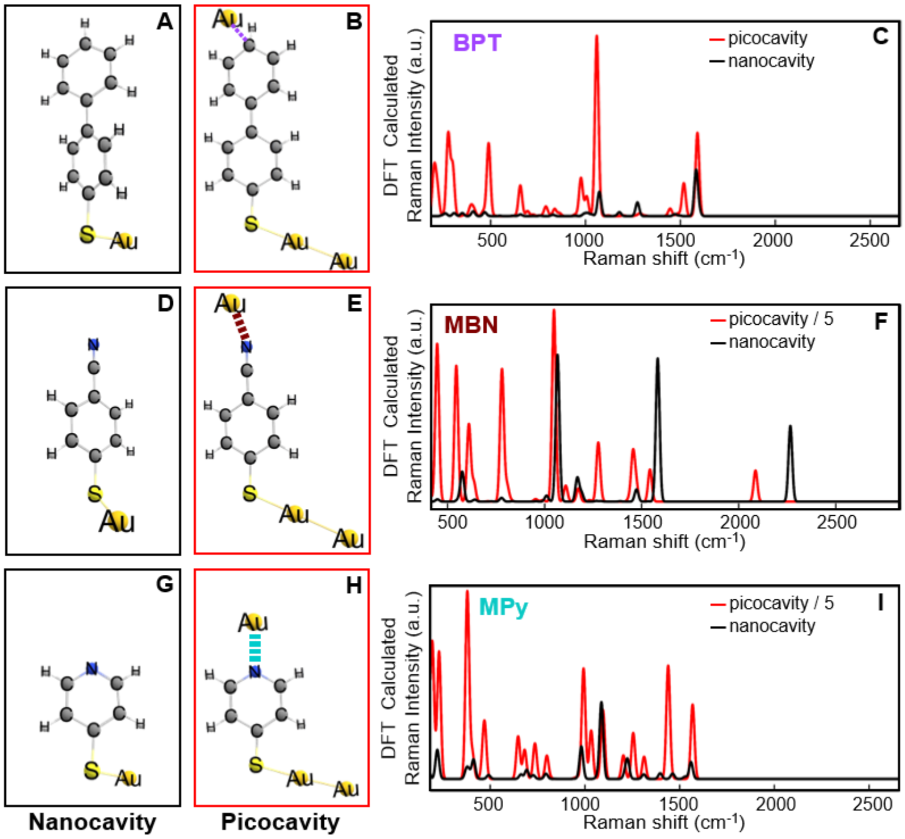

Fig. S3. Further density functional theory (DFT) calculations. (**A-C**) BPT. (**D-F**) MBN. (**G-I**) MPy. Left: Optimised molecular structures from calculations. Right: Comparison of DFT Raman spectra for the nanocavity (black, as (**A**), (**D**), (**G**)) and picocavity with the relative position of Au adatom from the minimum energy confirmation (red, (**B**), (**E**), (**H**)). In both nanocavity and picocavity, the charge state is 0 and the spin state is a singlet. In each picocavity, the Au adatom on top is $Au^{(0)}$. The bottom thiol is attached to two Au atoms to ensure an even number of electrons (otherwise attaching to one Au atom changes the system to a doublet, introducing undesirable impacts making comparison impossible). Note in (**F**) and (**I**), picocavity intensities are divided by 5, to be comparable with the nanocavity intensities on the same scale.

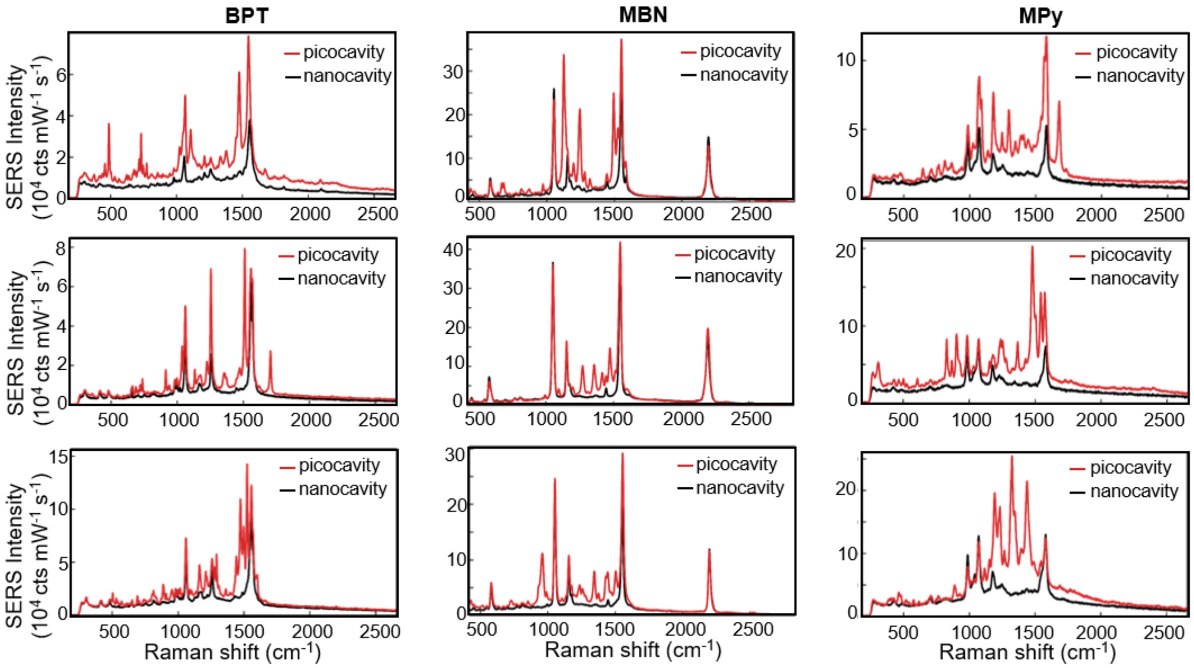

Fig. S4. Further picocavity SERS spectra examples (red). Variations and dynamics of picocavities due to different relative positions of Au adatom to nearby single molecule (see below Fig. S9), compared to the stable nanocavity SERS spectra (black).

Supplementary Note S2. Identification of picocavities and flares

The extraction of picocavities and flares is based on using a spectral dissimilarity metric of normalized Euclidean distance (ED),(*36*) while the segmentation uses a support vector machine (SVM) supervised machine learning model. The detailed steps are as follows:

1) Time dependent SERS spectra (Fig. S5A) are converted to dissimilarity series using their normalized ED distance between successive spectra x_i,y_i:

$$\mathrm{ED}(x, y) = \sqrt{\sum_{i=1}^{n} \frac{(x_i - y_i)^2}{\sqrt{x_i^2 + y_i^2}}} \qquad \text{(S1)}$$

As shown in Fig. S5B, the flares induce much higher ED distance variation than picocavities which thus allows their separation. The background of the time-dependent dissimilarity shows the intrinsic fluctuation of these time dependent SERS series, and is subtracted by an iterative polynomial method. The background-subtracted time-dependent dissimilarity of several clean SERS scans (without picocavities or flares) are selected for calculating the lower threshold for picocavities, threshold$_p$ = 2 x mean + 8 x (standard deviation). The lower flare threshold is obtained by comparing the ED values of flares (ED$_f$) and picocavities (ED$_p$) with the same spectral intensity, giving threshold$_f$ = threshold$_p$ x(ED$_f$/ED$_p$). A second flare threshold is set at double the first flare threshold to confidently catch higher intensity flares.

2) Fluctuations above the first picocavity threshold (see Fig. S5B, orange dashed line) and below the first flare threshold (see Fig. S5B, green dashed line) are identified as picocavity events. Those higher than the second flare threshold (see Fig. S5B, red dashed line) are identified as flare events. For the mixed region

(between first and second flare thresholds), the SERS spectra of each event are averaged, and the SERS background from the average spectrum is extracted and subtracted using an iterative polynomial method. After this the ED distances are recalculated to examine the contributions of sharp Raman peaks and smooth emission background. Depending on which contributes most to the dissimilarity variation, the event is assigned as a picocavity or flare. If both contribute, the event is considered as containing both picocavity and flare components.

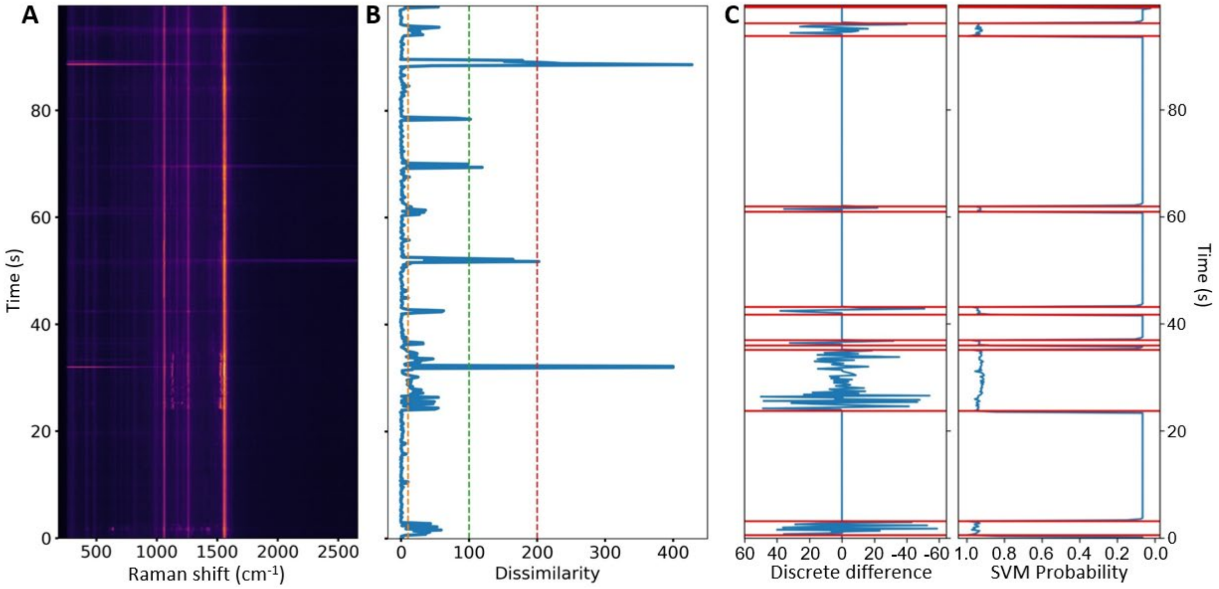

Fig. S5. Identification of picocavities and flares from time dependent SERS scans. (**A**) A time-series SERS spectra of BPT. (**B**) Dissimilarity of the $\boldsymbol{n}^{\text{th}}$ and $(\boldsymbol{n}\text{-}1)^{\text{th}}$ spectrum with time. Dashed lines show thresholds for separating picocavites and flares: first picocavity threshold (orange), first (green) and second (red) flare thresholds. (**C**) $\boldsymbol{n}^{\text{th}}$ discrete difference (left side) of the dissimilarity in b after removing flare events, and their support vector machine (SVM) probability (right side).

3) The events (picocavities or flares) separated in step 2 are segmented individually, in order from high to low threshold. Each trapped event is then removed from the dissimilarity series to avoid affecting the further segmentation of other regions. For performing the segmentation, dissimilarities below the threshold are set to 0 and time dependent dissimilarities converted to an nth discrete difference in time, as shown in Fig. S5C (this example segments events above the first picocavity threshold but below the first flare threshold). A semi-supervised approach using a support vector machine (SVM) model (with code in the open-source python library of pyAduioAnalysis (*48*)) is first trained with the discrete difference series, and then the SVM classifier is applied to output a probability series (giving confidence levels, see Fig. S5C right side). Finally, the probability series are used for separating the different event fluctuations with dynamic thresholding (see Fig. S5C, red lines).

Supplementary Note S3. Formation rate of picocavities and flares

Formation rates (P, in [s^{-1}]) can be extracted from the fraction of empty time-series where picocavities or flares are not observed (P_{empty}).

$$\left(P_{\text{empty}}\right)^{\Gamma} = \frac{n_{\text{empty}}}{n_{\text{time-series}}} \quad \text{(S2)}$$

$$(1-P)^{\Gamma} = \frac{n_{\text{empty}}}{n_{\text{time-series}}} \quad \text{(S3)}$$

$$P = 1 - \left(\frac{n_{\text{empty}}}{n_{\text{time-series}}}\right)^{1/\Gamma} \quad \text{(S4)}$$

where $n_{\text{time-series}}$ is the total number of time-series, n_{empty} is the number of empty time-series (without seeing any picocavities or flares over the time-series duration Γ). P is the probability of picocavity or flare formation, which is converted to formation rate.

Formation rates obtained by are used for Fig. 3C**,** and summarised in Table S1-2. Note that no empty time-series are seen for BPT at 255 $\mu\text{W}\ \mu\text{m}^{-2}$ meaning its flare formation rate (P_f) cannot be generated this way. Additional data from BPT-Br is plotted in Fig. S6 confirming validity of the fit from the main text:

$$P_p = P_{0p} \exp\{-U_f(I)/k_B T\} \quad \text{(S5)}$$

with $U_f(I) = U_f^0/(I/I_t + 1)$.

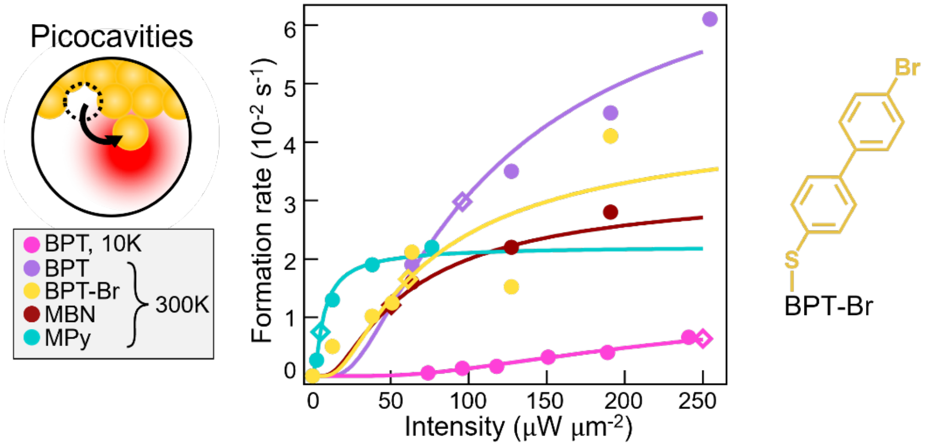

Fig. S6. Formation rate of four different molecules. Additional BPT-Br data is extracted from 758,500 spectra recorded in 1,517 time-series.

Table S1. Statistics of picocavities and flares across a range of laser power at room temperature, T=300 K. $n_{\text{time-series}}$ is the total number of time-series, Γ is duration of each time-series, n_{spectra} is the total number of spectra. N_p and N_f are the number of picocavities and flares, respectively. $n_{p,\text{empty}}$ and $n_{f,\text{empty}}$ are the number of empty time-series (without seeing any event over Γ). P_p and P_f are the formation rate of picocavities and flares, respectively.

BPT

I [μW μm^{-2}]	$n_{\text{time-series}}$	Γ [s]	n_{spectra}	N_p	N_f	$n_{p,\text{empty}}$	$n_{f,\text{empty}}$	P_p [10^{-2} s^{-1}]	P_f [10^{-2} s^{-1}]
0	N/A							0	0
64	346	100	173000	1734	582	50	46	1.9	2.0
127	327	100	163500	3335	904	9	26	3.5	2.5
191	394	100	197000	4313	1240	4	12	4.5	3.4
255	542	100	271000	6721	2690	1	0	6.1	N/A

MBN

I [μW μm^{-2}]	$n_{\text{time-series}}$	Γ [s]	n_{spectra}	N_p	N_f	$n_{p,\text{empty}}$	$n_{f,\text{empty}}$	P_p [10^{-2} s^{-1}]	P_f [10^{-2} s^{-1}]
0	N/A							0	0
64	119	120	14280	453	63	17	70	1.6	0.44
127	119	120	14280	553	110	8	44	2.2	0.83
191	119	120	14280	653	140	4	42	2.8	0.86

MPy

I [μW μm^{-2}]	$n_{\text{time-series}}$	Γ [s]	n_{spectra}	N_p	N_f	$n_{p,\text{empty}}$	$n_{f,\text{empty}}$	P_p [10^{-2} s^{-1}]	P_f [10^{-2} s^{-1}]
0	N/A							0	0
3	59	180	10620	51	17	36	43	0.27	0.18
13	59	180	10620	291	52	6	20	1.3	0.60
38	59	180	10620	379	53	2	17	1.9	0.69
76	59	180	10620	424	57	1	21	2.2	0.57

Table S2. Statistics for formation rate fitting constant ($P_{p0,p}$ and $P_{p0,f}$) and power threshold for both picocavities and flares (I_c).

	**BPT**	**MBN**	**MPy**	**BPT** (*19*)
T [K]	300 K	300 K	300 K	10 K
$P_{p0,p}$ [10^{-2} s^{-1}]	8.2	3.3	2.2	1.8
$P_{p0,f}$ [10^{-2} s^{-1}]	6.2	1.2	0.73	N/A
I_c [μW μm^{-2}]	97	51	5.5	263

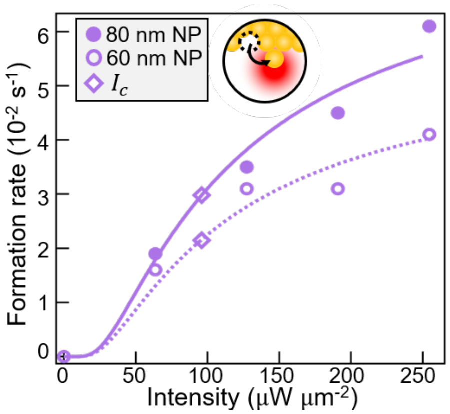

Fig. S7. Formation of picocavities for BPT, for 80 nm *vs* 60 nm NPs. Additional 60 nm NPs data is extracted from 382,500 spectra recorded in 765 time-series from 114 NPoMs. 80 nm NPs data is from Fig. 3C. Lines are fits, ◇ shows critical intensity, which is the same for both sizes.

Supplementary Note S4. Decay rate of picocavities and flares

The lifetime (T, in [s]) of each transient event is modelled with a biexponential probability density function (PDF)

$$P(t)dt = \left[W\, r_{fast}\, e^{-r_{fast}t} + (1-W)\, r_{slow}\, e^{-r_{slow}t}\right]dt \tag{S6}$$

where r_{fast} and r_{slow} (in [s^{-1}]) are the decay rates, and f_{fast} and f_{slow} (in [%]) are the fractions of fast and slow events. Here t is event length in units of the integration time per spectrum and the PDF is parameterised by $0 \le W \le 1, r_{fast} > 0, r_{slow} > 0$. Experimentally, lifetimes can only be measured in integer multiples of the integration time. The probability of measuring an event lasting n integration times is

$$P_n \propto \int_{n-\frac{1}{2}}^{n+\frac{1}{2}} P(t)dt \tag{S7}$$

where the constant of proportionality is set to normalise the discrete probability distribution. We now perform maximum log-likelihood estimates of the measured set of lifetimes $N = \{n_i\}$ by defining

$$L = \sum_i \ln(P_{n_i}) \tag{S8}$$

The most likely values for the parameters W, r_{fast}, r_{slow} are those that maximise the log-likelihood L of measuring the observed values. Uncertainty bounds are placed on these values using the standard result of altering the parameter until L drops by $\frac{1}{2}$ from its maximised value. The extracted $r_{\text{fast}}, r_{\text{slow}}, f_{\text{fast}}$ and f_{slow} are summarised in **Tables S3,4**.

We note that the number of fast flares seen for MBN and MPy are too small to give reliable fits to histogram decay rates. The single exponential probability density function is used to perform maximum log-likelihood estimates of each lifetime for slow decay rates.

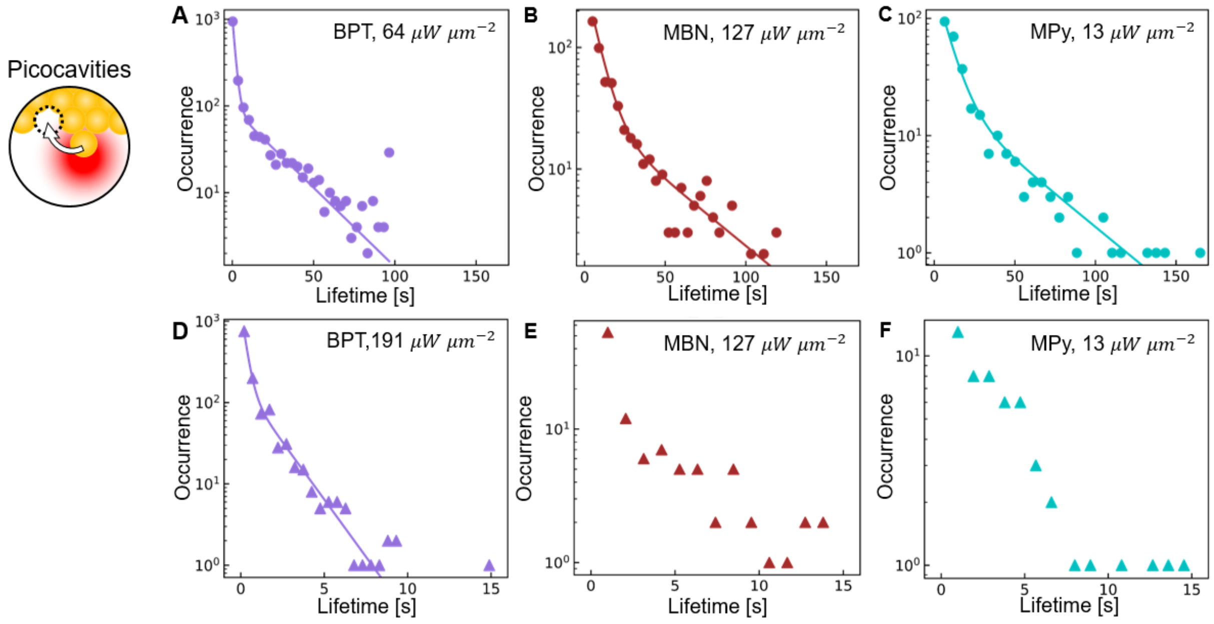

Fig. S8. Exemplar lifetime distributions. Occurrence distributions recorded vs lifetime at different laser intensity for picocavities (**A**-**C**) and flares (**D**-**F**). The log scale plot shows the biexponential distributions (except for flares in MBN and MPy where fast flares seen are too infrequent to give reliable fits and single exponential PDF used to extract slow decay rates). The biexponential PDF is used for the other parameter extractions.

Table S3. Statistics of decay rate (r_{fast} and r_{slow}) and fraction (f_{fast} and f_{slow}) of fast and slow picocavities.

BPT

$I\ [\mu W\ \mu m^{-2}]$	f_{fast} [%]	$r_{\text{fast}}\ [10^{-2}\ s^{-1}]$	f_{slow} [%]	$r_{\text{slow}}\ [10^{-2}\ s^{-1}]$
64	$59.6^{+1.4}_{-1.5}$	$50.9^{+2.3}_{-2.2}$	$40.4^{+1.5}_{-1.4}$	$3.59^{+0.15}_{-0.14}$
127	$72.8^{+1.0}_{-1.0}$	$47.8^{+1.4}_{-1.3}$	$27.2^{+1.0}_{-1.0}$	$5.37^{+0.20}_{-0.19}$
191	$77.6^{+0.9}_{-0.8}$	$48.3^{+1.1}_{-1.1}$	$22.4^{+0.8}_{-0.9}$	$5.63^{+0.20}_{-0.20}$
255	$69.2^{+0.8}_{-0.8}$	$51.4^{+1.1}_{-1.0}$	$30.8^{+0.8}_{-0.8}$	$7.64^{+0.19}_{-0.18}$

MBN

$I\ [\mu W\ \mu m^{-2}]$	f_{fast} [%]	$r_{\text{fast}}\ [10^{-2}\ s^{-1}]$	f_{slow} [%]	$r_{\text{slow}}\ [10^{-2}\ s^{-1}]$
64	43^{+4}_{-4}	$11.7^{+1.4}_{-1.3}$	57^{+4}_{-4}	$2.62^{+0.18}_{-0.17}$
127	53^{+4}_{-4}	$11.3^{+1.1}_{-1.0}$	47^{+4}_{-4}	$3.25^{+0.22}_{-0.22}$
191	77^{+3}_{-3}	$10.2^{+0.6}_{-0.6}$	23^{+3}_{-3}	$3.15^{+0.3}_{-0.29}$

MPy

$I\ [\mu W\ \mu m^{-2}]$	f_{fast} [%]	$r_{\text{fast}}\ [10^{-2}\ s^{-1}]$	f_{slow} [%]	$r_{\text{slow}}\ [10^{-2}\ s^{-1}]$
3	38^{+10}_{-10}	14^{+5}_{-4}	62^{+10}_{-10}	$1.5^{+0.3}_{-0.3}$
13	69^{+5}_{-6}	$8.4^{+0.9}_{-0.8}$	31^{+6}_{-5}	$2.7^{+0.3}_{-0.3}$
38	58^{+6}_{-6}	$9.6^{+1.1}_{-1.0}$	42^{+6}_{-6}	$3.5^{+0.3}_{-0.3}$
76	53^{+8}_{-8}	$8.5^{+1.0}_{-0.9}$	47^{+8}_{-8}	$4.3^{+0.3}_{-0.3}$

Table S4. Statistics of decay rate (r_{fast} and r_{slow}) and fraction (f_{fast} and f_{slow}) of fast and slow flares.

BPT

I $[\mu W\ \mu m^{-2}]$	f_{fast} [%]	r_{fast} $[10^{-2}\ s^{-1}]$	f_{slow} [%]	r_{slow} $[10^{-2}\ s^{-1}]$
64	$98.3^{+1.1}_{-1.6}$	126^{+6}_{-6}	$1.7^{+1.6}_{-1.1}$	39^{+19}_{-13}
127	$94.7^{+2.0}_{-2.3}$	108^{+4}_{-4}	$5.3^{+2.3}_{-2.0}$	42^{+9}_{-7}
191	$86.2^{+2.2}_{-2.1}$	118^{+5}_{-5}	$13.8^{+2.1}_{-2.2}$	41^{+4}_{-3}
255	$74.9^{+2.2}_{-2.1}$	125^{+4}_{-4}	$25.1^{+2.1}_{-2.2}$	55^{+2}_{-2}

MBN

I $[\mu W\ \mu m^{-2}]$	f_{fast} [%]	r_{fast} $[10^{-2}\ s^{-1}]$	f_{slow} [%]	r_{slow} $[10^{-2}\ s^{-1}]$
64	N/A	N/A	N/A	26^{+3}_{-3}
127	N/A	N/A	N/A	$20.2^{+2.0}_{-1.9}$
191	N/A	N/A	N/A	$20.2^{+1.8}_{-1.7}$

MPy

I $[\mu W\ \mu m^{-2}]$	f_{fast} [%]	r_{fast} $[10^{-2}\ s^{-1}]$	f_{slow} [%]	r_{slow} $[10^{-2}\ s^{-1}]$
3	N/A	N/A	N/A	29^{+8}_{-6}
13	N/A	N/A	N/A	25^{+4}_{-3}
38	N/A	N/A	N/A	27^{+4}_{-3}
76	N/A	N/A	N/A	28^{+4}_{-3}

Supplementary Note S5. Molecule-metal coordination bonds

We estimate the molecule-$Au^{(0)}$ adatom interaction energies for MPy, BPT, and MBN systems with a series of constrained DFT optimizations. In these calculations the molecules are attached to two gold atoms via their thiol groups, and a third gold atom (adatom) close to the N atom of MPy and MBN, and the C atom of BPT (see **Fig. S3,S9**). The adatom positions are defined at grid points on a plane perpendicular to the aromatic rings of MBN and MPy, or perpendicular to the upper aromatic ring of BPT. To fix the adatom with respect to the molecule, the N/C-adatom distance (z in **Fig. S9**, 1.8-3.2 Å, with 0.1 Å increments) and angle between the adatom, the N/C atom and a third atom (θ in **Fig. S9** with 10° increments), were fixed. For each series of calculations, we fit a Morse potential curve along the line passing through the lowest energy (at fixed θ, black dashed in **Fig. S9A**):

$$E_{rel} = U_b^0 \cdot \left[1 - \exp\left(-a \cdot (z - z_0)\right)\right]^2 \qquad \text{(S9)}$$

with z_0 and U_b^0 giving the position and depth of the minimum. These parameters are summarized in **Table S6A**. In **Fig. S11A**, the fitted Morse potential curves are overlain to show that the same BPT<MBN<MPy order is observed for the interaction energies as for the induced charges in the presence of an external electric field (**Fig. 5,S17**). The calculations are repeated for molecule-$Au^{(I)}$ coordination bonds (**Table S6b**, **Fig. S11B)**. This is to show that even with very rare cases when $Au^{(I)}$ is formed on nanoparticles due to lattice strain, the binding energies follow the same BPT<MBN<MPy order as for molecule-$Au^{(0)}$.

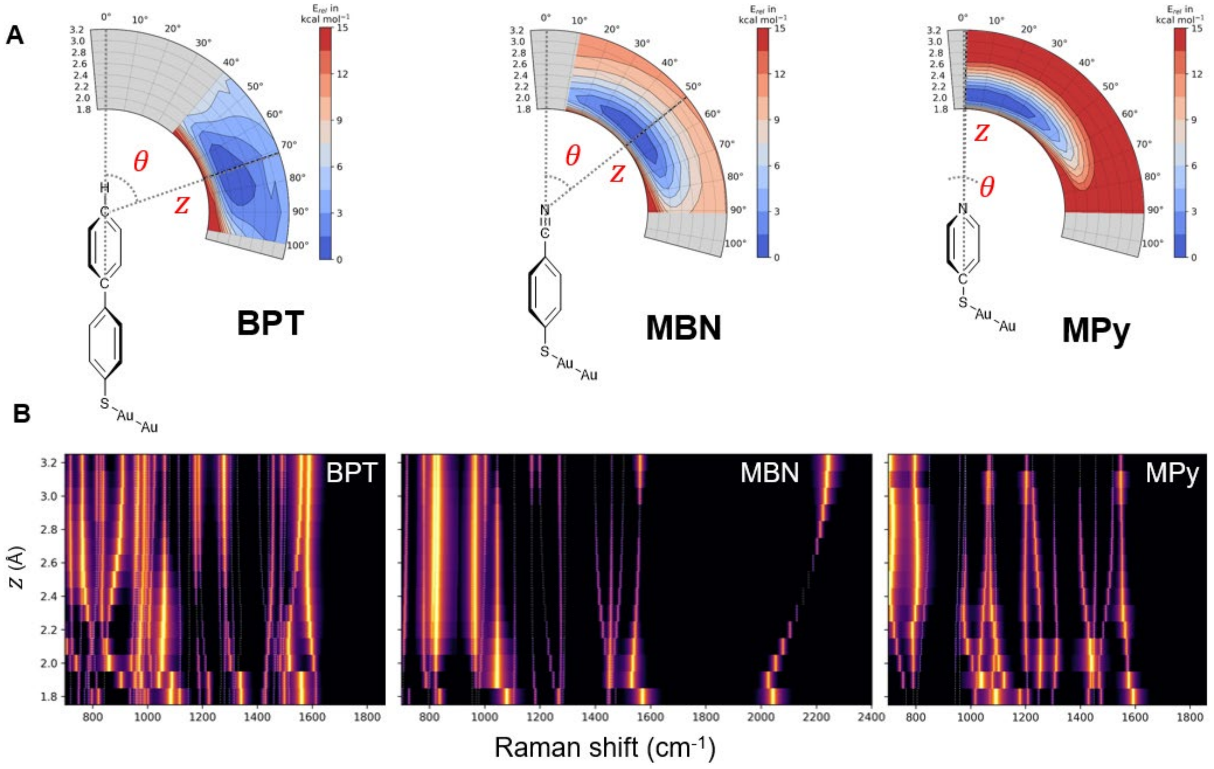

Fig. S9. Molecule-adatom conformations varying adatom position relative to tip of molecule. (**A**) Relative electronic energies for each molecule at conformations defined by $\boldsymbol{z}$, $\boldsymbol{\theta}$ ($\boldsymbol{\varphi}$ not shown) on the depicted planes perpendicular to the aromatic ring of BPT, MBN, and MPy (upper ring). Black dashed lines mark lowest energy, with Morse potential curves fit along these lines. (**B**) DFT calculated picocavity Raman spectra, decreasing adatom spacing from tip of molecule ($\boldsymbol{\theta}$,$\boldsymbol{\varphi}$ fixed) showing spectral shifts in vibrational peak positions. This confirms picocavity spectra depend critically on the 1 nm^3 confined optical field and relative position of Au adatom to nearby single molecule.

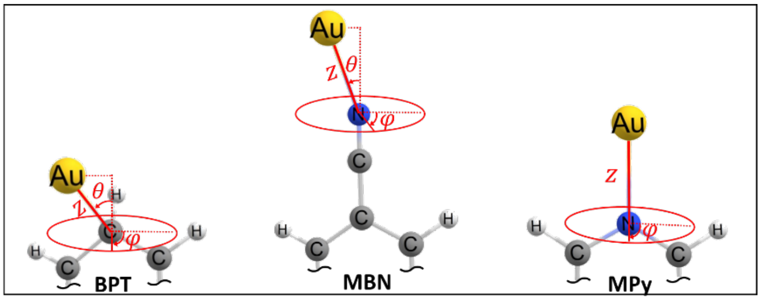

Fig. S10. Coordinate system for Au adatom position. $\boldsymbol{\varphi}$ defined as shown

Table S5. Optimised Au adatom positions found by DFT calculations.

	BPT	MBN	MPy
θ (°)	75	49	0
φ (°)	91	90	91
z (Å)	2.2	2.2	2.2

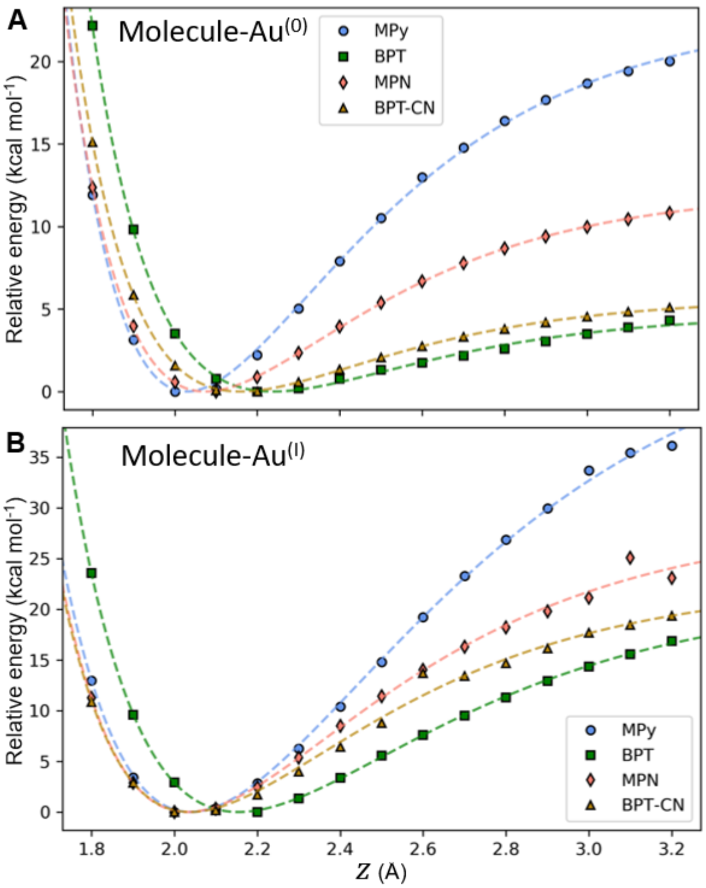

Fig. S11. Fitted Morse potential curves. Relative energies *vs* $\boldsymbol{z}$ along the black dashed lines in Fig. S9A. Dashed lines are the Morse potential curves fitted to each dataset. (**A**) Molecule-Au$^{(0)}$ adatom, and (**B**) Molecule-Au$^{(I)}$ adatom.

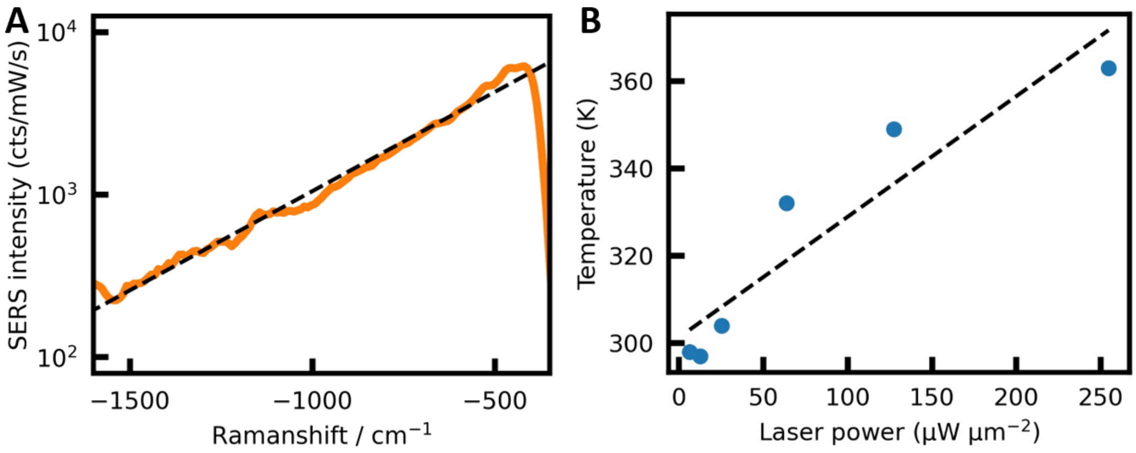

Fig. S12. Laser power heating of NPoMs. (**A**) Example anti-stokes SERS spectrum at 250 μW μm^{-2} on log scale together with exponential fit for extracting temperature (dashed line). (**B**) Temperature from anti-stokes SERS vs laser power. Maximum temperature increase is 70 K at 250 μW μm^{-2} corresponding to an energy scale of ~ 6 meV.

Table S6. Parameters obtained from fitting Morse potential curves to the relative energies correspond to the points along the black dashed lines in Fig. S9A. (**A**) Molecule-Au(0), and (**B**) Molecule-Au(I) adatom.

	Molecule	U_b^0 (kcal mol^{-1})	U_b^0 (eV)	a (Å^{-1})	z_0 (Å)
**A**	BPT	4.75	0.21	2.63	2.24
	BPT-CN (*32*)	5.67	0.25	2.70	2.16
	MBN	12.20	0.53	2.55	2.07
	MPy	23.01	1.00	2.38	2.03
**B**	Molecule	U_b^0 (kcal mol^{-1})	U_b^0 (eV)	a (Å^{-1})	z_0 (Å)
	BPT	21.53	0.93	2.02	2.16
	BPT-CN (*32*)	22.69	0.98	2.22	2.04
	MBN	28.84	1.25	2.09	2.03
	MPy	49.78	2.16	1.73	2.04

Supplementary Note S6 | Discussion of optical forces near picocavities

A successful model in the literature (*15*) provided by full time-dependent DFT of the optical field around a picocavity Au adatom appears to match well that from a metallic sphere (permittivity ϵ_m) of the size of an atom (radius a) in the uniform field $\mathcal{E}_g$ of the nanocavity gap (permittivity ϵ_g). The field is concentrated at the tip of the atom (see below), in a field distribution which looks like that from a dipole positioned at the atom centre (Fig. S14).

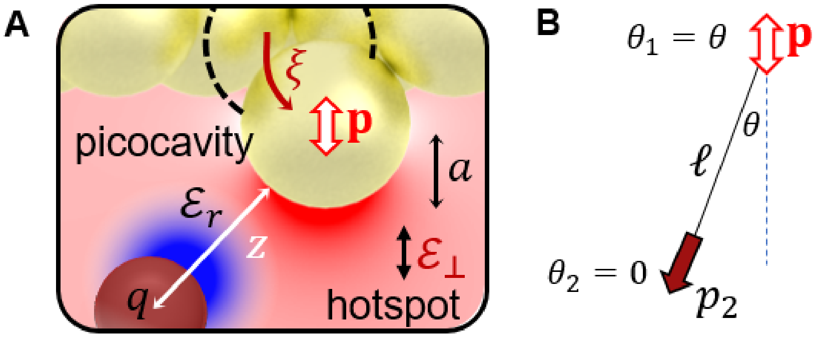

Fig. S13. Picocavity induced dipoles. (**A**) Dipolar field distribution from light at picocavity (red) giving induced charge $\boldsymbol{q}$ on tip atom (brown) of molecule with perturbed outer electronic orbitals (blue). (**B**) Optical dipole $\mathbf{p}$ produced by illumination induces a dipole $\boldsymbol{p_t}$ at the tip atom of the molecule.

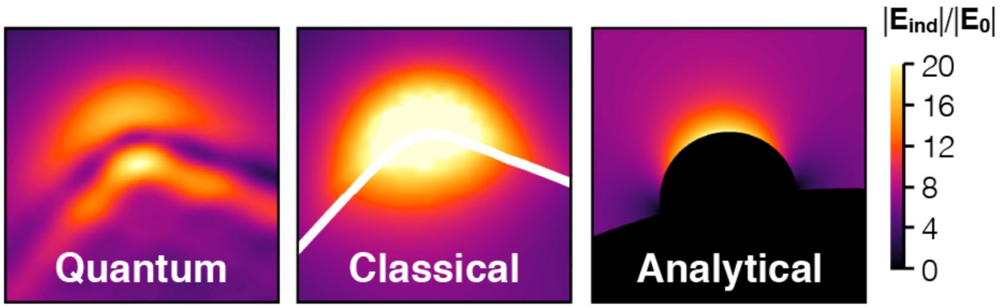

Fig. S14. Picocavity fields around single atom. Comparison of quantum, classical, and analytical optical field distributions for a picocavity (*15*). Figure used with permission from (*15*).

The field at radial coordinates $(r = a + \delta, \theta)$ of a dipole $\boldsymbol{p}$ at the origin is given by

$$\mathcal{E} = \mathcal{E}_g + \frac{1}{4\pi\epsilon_0}\left(\frac{3(\boldsymbol{p}.\hat{\boldsymbol{r}})\hat{\boldsymbol{r}} - \boldsymbol{p}}{r^3}\right)$$

Ensuring that there is no field parallel to the metal sphere surface (so $\boldsymbol{p}.\hat{\boldsymbol{r}}$=0), we get

$$\boldsymbol{p} = 4\pi\epsilon_0 a^3 \mathcal{E}_g \hat{z}$$

For long wavelengths where the metal is very good this is correct. There is a modification to this for smaller $|\epsilon_m|$ (i.e. not perfect metals):

$$\mathcal{E} = \mathcal{E}_g + \frac{1}{4\pi\epsilon_0}\left(\frac{3\aleph(\boldsymbol{p}.\hat{\boldsymbol{r}})\hat{\boldsymbol{r}} - \boldsymbol{p}}{r^3}\right), \quad \text{with} \quad \aleph = -\frac{1}{3}\frac{1}{L + [\epsilon_m/\epsilon_g - 1]^{-1}}$$

where L codes for the shape of the protrusion and is 1/3 for a sphere.(*49*)

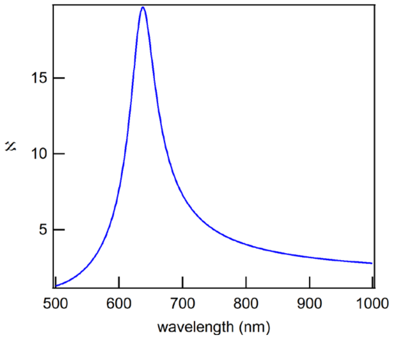

Fig. S15. Local field enhancement ℵ near metal of specific asperity (here for a sphere above surface).

In this model, the extra enhancement ℵ reaches 20 for an aspect ratio of the picocavity of 2:1 (i.e. with the Au atom perched on top of another atom, completely out of the facet), and is 3.6 for the hemisphere drawn in **Fig. S14**. It also depends on wavelength.

The field outside the metal sphere in (x, z) coordinates is then (*50*)

$$\mathcal{E} = \mathcal{E}_g + a^3\mathcal{E}_g\left(\frac{3\aleph\cos\theta\,(\sin\theta, \cos\theta) - (0,1)}{(\delta + a)^3}\right)$$

or

$$\frac{\mathcal{E}}{\mathcal{E}_g} = \binom{0}{1} + (1 + \delta/a)^{-3}\left[3\aleph\cos\theta\binom{\sin\theta}{\cos\theta} - \binom{0}{1}\right]$$

Comparison to the TDDFT and classical solutions of the field around the picocavity matches this well.(*15*)

Supplementary Note S7. Total Electrostatic Energy

To evaluate the increase in energy when moving out this conducting sphere into the uniform gap field, we evaluate (outside the sphere, and just for the top half space)

$$U \simeq \int dV\, \tfrac{1}{2}\epsilon_0\epsilon\left(|\mathcal{E}|^2 - |\mathcal{E}_g|^2\right) \qquad \text{(S10)}$$

where for $\delta' = \delta/a$, the explicitly calculated term

$$|\mathcal{E}|^2 - |\mathcal{E}_g|^2 = |\mathcal{E}_g|^2\{[1 + (1 + \delta')^{-3}(3\aleph\cos^2\theta - 1)]^2 + [(1 + \delta')^{-3}3\aleph\cos\theta\sin\theta]^2 - 1\}$$

giving after some algebra

$$(|\mathcal{E}|^2 - |\mathcal{E}_g|^2)/|\mathcal{E}_g|^2 = 2(1 + \delta')^{-3}(3\aleph\cos^2\theta - 1) + (1 + \delta')^{-6}(3\aleph\cos^2\theta + 1)$$

hence integrating over the volume gives:

$$U = \epsilon_0 \epsilon \left|\mathcal{E}_g\right|^2 \aleph' \pi a^3 \frac{2}{3}$$

with $\aleph' = (1+\aleph)/2 \sim 2$ near the resonance condition.

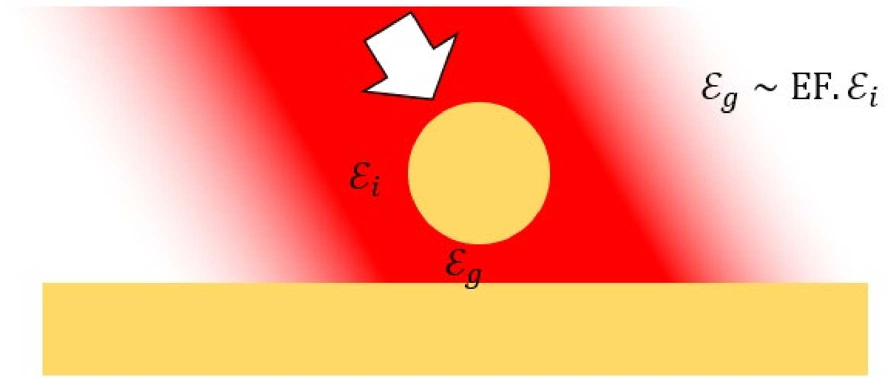

Fig. S16. Illumination of NPoM, parametrising the field $\boldsymbol{\mathcal{E}_i}$ in which the NP is bathed, and the enhanced field $\boldsymbol{\mathcal{E}_g}$ in the gap.

For an incident intensity $I = \frac{1}{2} c\epsilon_0 |\mathcal{E}_i|^2$, which is coupled into the NPoM with efficiency η and nanocavity field enhancement factor EF we have

$$U = \eta \frac{2I}{c} \mathrm{EF}^2 \, \aleph' \pi a^3 \frac{2}{3} = \eta \frac{I}{c} \aleph' \, \mathrm{EF}^2 \, a^3 \frac{4\pi}{3} \qquad \text{(S11)}$$

For an intensity of 1 mW/µm^2, n =1.5, a =0.14nm (for Au), EF =1000, and η =0.4, we get energies $U \simeq$ 0.6meV. As the Au adatom size decreases, **Eq. S11** shows the energy decreases because the dipole size gets smaller.

We note that the simple energy estimate here misses a contribution due to optical field expelled from the metal when the picocavity exists (as seen in TDDFT simulations). This can be understood from the much higher in-plane $k_{\|}$ required to localise light around the single atom, which gives a much higher imaginary out-of-plane $k_{\perp}$, and thus short decay length into the metal set by the gap size d.(*28*) The energy from this expulsion is estimated as

$$U_{expel} \sim (\pi a^2) \left(\frac{d\epsilon_m}{2\epsilon_g}\right) \tfrac{1}{2} \epsilon_0 \epsilon_m \left(\frac{\epsilon_g}{\epsilon_m} \mathrm{EF}.\mathcal{E}_g\right)^2 = \frac{\pi}{2} a^2 d \epsilon_g \mathrm{EF}^2 \frac{I}{cn} \sim \frac{I}{c} \mathrm{EF}^2 a^2 d$$

This rough estimate suggests a larger energy for the picocavity field, by a factor up to d/a~10. A more detailed calculation at the single atom level is thus required but is currently beyond both classical theory and DFT, for instance to include contributions within the metal from terms in $\frac{\partial}{\partial\omega}\{\epsilon_m\}$.(*51*)

Supplementary Note S8. Optical Forces

We now discuss the models that are most plausible for understanding how only 1-10 µW/µm^2 of incident CW light is capable of pulling a Au adatom out of the facet, which the experimental data shows. The rectification of the optical frequency fields produces a gradient force $\propto \nabla\mathcal{E}^2$ that acts on the atoms in the facet.(*52*)

To understand the scale of forces required, we note that for the typical Au adatom barrier energy of 1eV (*25*, *26*, *53*) when moving a Au atom out by the 0.1 nm distance needed to create the intense picocavity hotspot would require that $F_0 \simeq \Delta U/\Delta r \simeq 1e/10^{-10}$~2 nN is provided. We show below it is hard to directly develop this force using the intensities used in our experiments. We also note adatom surface diffusion barriers are of similar scale.(*54*)

The model explored here in most detail is a **field-plucking model** in which the light locally polarizes the tip of the molecule to enhance the force between it and the Au atom (via rectified *dc* optical forces). One other model more carefully considered below and discarded is the **hot atom model** in which all the photon energy is given to one Au atom (instead of a hot electron) which can now escape over the barrier.

Field-plucking model

Considering that the total energy (**Eq. S11**) is produced by moving the adatom out by a distance a (the radius of the atom), we get (as our **Model 1**)

$$F \simeq -\frac{dU}{dr} = \eta \frac{I}{c} \aleph' \, \mathrm{EF}^2 \, a^2 \frac{4\pi}{3} \tag{S12}$$

This is equivalent to the optical power density (I/c) exerted on an area πa^2 and enhanced by the optical intensity in the nanocavity, and for the values above comes to 1 pN per mW of tightly focussed incident light. This is thousands of times smaller than F_0 for the intensities used in our experiments. It thus appears to be much too small to explain why we can pull out picocavities with intensities of 10 µW or less (for MPy), and 50 µW for BPT.

We consider possible enhancements to the optical force from:

Induced local molecular dipole: produced by the picocavity optical dipole field which induces changes in the charge distribution of the nearby molecule. The nearby electric dipole induced by the light perturbs the molecular orbitals (both the charge that they contain, and also possibly their shape). Effects can arise both from the local field at the atoms, as well as the field gradient at the atoms (since E varies on the scale of the atom from **Note S6**). Full DFT calculations allow us to estimate these perturbations.

Initially we make an approximation that such changes can be localised as an induced point dipole p_t at the core position (at distance ℓ) of the nearest atom to the Au (since the picocavity field decays so fast spatially),

$$p_2 = \beta \epsilon_0 \mathcal{E}(\ell) + \gamma \epsilon_0 \, \nabla \mathcal{E}(\ell)$$

Ideally it would be possible to identify β, γ from DFT, but separating them is challenging since the influence of the optical dipole nearby produces both $E, \nabla E$ at the atomic site. Whilst β is related to α_0, it measures the polarizability of the tip atom (α_t) and not the whole molecule (which gives α_0 in a uniform applied field).

Plasmonic dipole multiplication: This local dipole p_t (**Fig. S13**) then induces additional image dipoles in the nearby metal, and this creates a term which can become extremely large. A simple way to estimate this comes from assuming the final dipole p_t elicited at the atomic site is produced from the sum of the quasistatic field initially created there as well as the additional induced field $\mathcal{E}_{im}$ from image charges,(*38*)

$$\mathcal{E}_{im} = \frac{\aleph p_t}{4\pi\epsilon_0 (2z)^3}$$

where z is the distance of the dipole from the metal surface (as above). There will be changes in this from the curvature of the picocavity surface, and also from image charges in the other metal facet, but since this tip molecule atom is so close to the picocavity adatom, it can be a reasonable approximation. The dipole produced

$$p_t = \alpha \mathcal{E}_g = \alpha_t (\mathcal{E}_g + \mathcal{E}_{im})$$

then yields when solving for $\mathcal{E}_{im}$ the effective polarizability

$$\frac{\alpha}{\alpha_t} = \frac{1}{1 - (\alpha_t \aleph/8)/z^3} = \frac{1}{1 - \kappa^3/z^3} \equiv \zeta \tag{S13}$$

These image charges thus amplify the dipole produced at the atomic site, by a value that depends critically on its separation and polarizability. If we look at typical values of α_0 for HCN (2.6 Å^3), benzene (10 Å^3),

pyridine (9.5 Å^3), we see that $\kappa = (\alpha_0 \aleph/8)^{1/3}$ ~1.1Å for ℵ=1 and ~2.9Å for ℵ=20. For $d < \kappa$ the dipole becomes infinite in this model. This 'polarization catastrophe' is normally suggested to induce a perturbation that becomes so large that it breaks the molecule, or by deforming it switches on a permanent dipole when it is so close to the metal. It is thus plausible that the resulting force which is also now amplified, instead of breaking the molecule, now breaks the Au binding to the facet in order to pull out a picocavity.

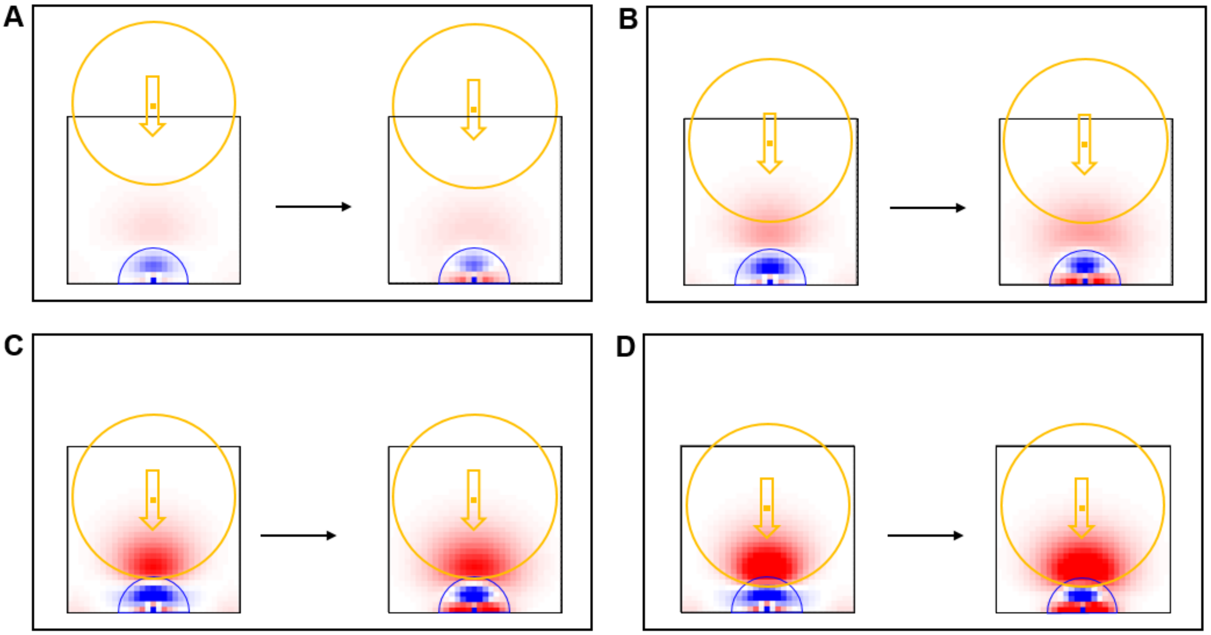

Fig. S17. Extraction of local tip atom polarizability induced. Charge distribution from DFT at the tip of MPy as the dipole approaches from the optimal $\boldsymbol{\theta}, \boldsymbol{\phi}$, at (**A**) $z = \mathbf{3.3}$ Å, (**B**) $\mathbf{2.6}$ Å, (**C**) $\mathbf{2.1}$ Å, and (**D**) $\mathbf{1.9}$ Å.

Local polarizability of molecule tip: While the above derivation uses the full α_0 in κ, this should be modified. Instead of applying a uniform field on the molecule to induce this dipole p_t (as conventionally calculated), here we need the local polarizability just on the molecule tip atom closest to the adatom. Thus instead we take $\kappa = (\beta\aleph/8)^{1/3}$ using values from DFT.

To estimate the induced dipole produced by a picocavity, we use DFT simulations of a Au adatom in different locations around the molecular tip (**Fig. S17**). We place a fixed dipole in these different positions (pointing towards the tip atom) and extract the induced charge shifts across the whole molecule. **Table S5** shows the stable positions of minimum energy found for the adatom-molecule system, using the coordinate system in **Fig. S10**. The induced charge on the tip (N or C) atom of the molecule is then extracted as a measure of the point dipole at this atom. The component of polarizability from a z-directed metal atom dipole is then the induced charge multiplied by $\cos^2\theta$ (for projection direction of field, and projection of vector polarizability). This is clearly seen to scale with the coordination strength of the bond (**Fig. S18B**) as well as the experimental critical intensity (**Fig. S18C**).

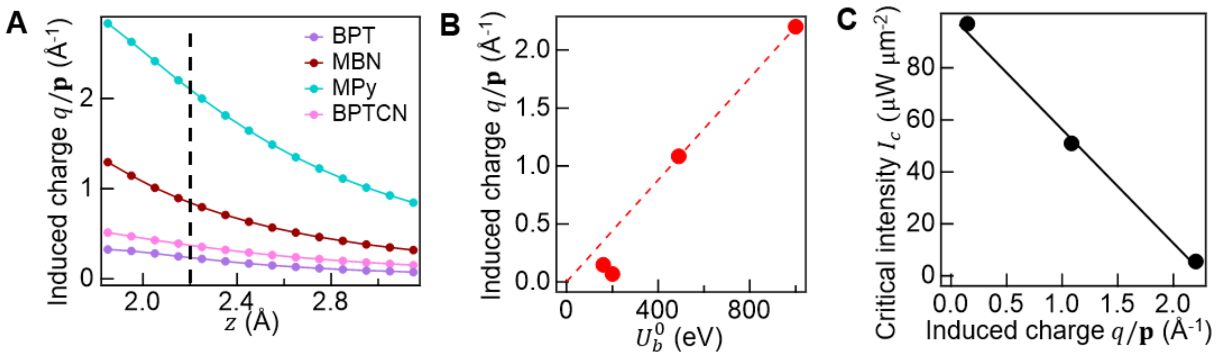

Fig. S18. Induced charge for dipole approaching tip of molecule. (**A**) Induced charge (normalised to excitation dipole on adatom), as the Au-N (or Au-C) separation is changed, for different molecules (taken along approach direction of minimum energy). (**B**) Correlation of this tip polarizability with the depth of the energy well for the coordination bond. (**C**) Critical intensity measured in experiments, compared to induced tip polarizability.

We now estimate the amplified force in this model. We take the dipole-dipole energy between the picocavity adatom dipole, and the induced dipole on the nearest atom (N or C) located at $\ell = a + z$,

$$U(r) = \frac{1}{4\pi\epsilon_0 \ell^3}[\mathbf{p}.\boldsymbol{p}_2 - 3(\mathbf{p}.\hat{\boldsymbol{r}})(\boldsymbol{p}_t.\hat{\boldsymbol{r}})]$$

hence assuming a radially-directed field $\mathcal{E}_r$ at the nearby atomic site

$$U = -\frac{\mathrm{p}}{2\pi\epsilon_0 \ell^3}\cos\theta\,[\beta\epsilon_0\mathcal{E}_r(\mathrm{p},\ell) + \gamma\epsilon_0\nabla\mathcal{E}_r(\mathrm{p},\ell)]\,\frac{1}{1-(\kappa/(\ell-a))^3}$$

Substituting and simplying as a function of $z = \ell - a$,

$$U = -2\epsilon_0\mathcal{E}_g^2\cos^2\theta\,\frac{1}{(1+z/a)^3}\left\{\beta\left[1+\frac{2}{(1+z/a)^3}\right] - \frac{\gamma}{a}\,\frac{6}{(1+z/a)^4}\right\}\,\frac{1}{1-(\kappa/z)^3}$$

Very near the metal surface z/a<1, so we can define $\varsigma = 2\cos^2\theta\,.\,a^{-3}\{3\beta - 6\gamma/a\,\}$ to obtain

$$U \sim -\,\epsilon_0\mathcal{E}_g^2 a^3\,\zeta\varsigma$$

The force that arises when gaining this energy by moving out the adatom by a to be next to the molecule is then (similar as in **Eq. S12**) given by

$$F \sim \eta\frac{2I}{c}\aleph'\,\mathrm{EF}^2 a^2\,\zeta\varsigma \qquad \text{(S14)}$$

Compared to the previous estimate of the force (**Eq. S12**), we now have the additional final term $\zeta\varsigma$ which diverges as the atom core approaches to the surface adatom within $d{\sim}\kappa{\sim}$1-2 Å (**Fig. S19**). Note that ζ (defined in **Eq. S13**) comes from the image dipole enhancement of the molecular tip dipole, and ς estimates the magnitude of this molecular tip dipole.

Including the local field (ignoring the $\nabla\mathcal{E}_r$ term), and provisionally taking $\beta = \alpha_0$, $\varsigma \sim 6a^{-3}\alpha_0 = 48(\kappa/a)^3$ with parameters for pyridine, giving the average forces shown in **Fig. S19**.

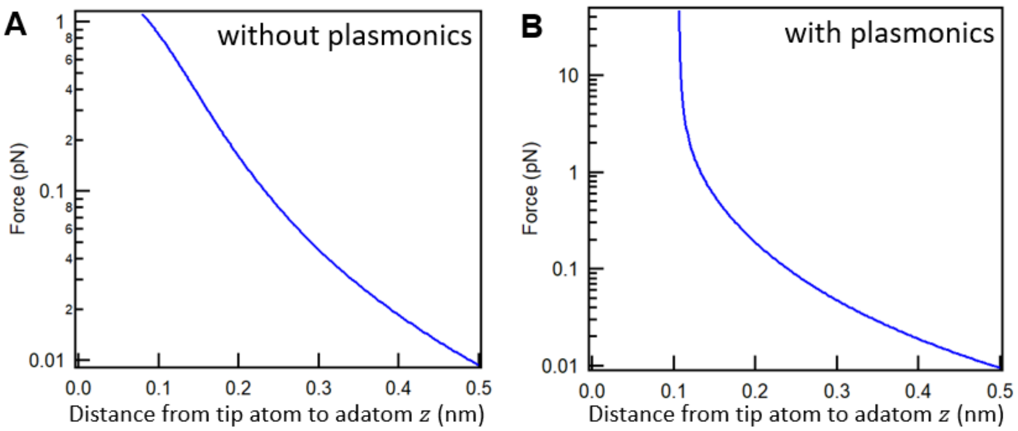

Fig. S19. Model plucking force including local polarizability and plasmonics. Optical force per 0.1 mW incident at a picocavity (**A**) without, and (**B**) with the extra contribution of image enhancements from the molecular tip dipole.

This suggests that picocavities can arise from the polarization divergence due to image charges at the facet surface. It creates such large forces (nN) that the Au atom is pulled from the facet, which then modifies the polarization divergence as discussed below. We note that the optical pressures focussed on the single atom here approach a million bar. Another way to consider this divergent polarizability is the local effective refractive index, which in this formalism approaches n_{loc}=10 for the molecule 0.2 nm from the facet in a 1 nm gap containing initial refractive index of 1.5. However we also stress that a full calculation needs to account for the interpenetration of the electron clouds on Au and tip atom (C or N) which smears out the divergence (as in the Jones-Jennings-Jepsen model (*55*)), as well as not using point dipoles but delocalised electrons and treating fully the screening electrons in the metal in a quantum model. Another issue is that the image plane position can be further out from the metal nuclear core by z_0=1.2Å. A full resolution of the forces awaits a theoretical treatment capable of treating quantum and classical contributions on the same footing. We note that molecules with lower local polarizability (such as alkanethiols) can never reach this polarization divergence regime and so indeed are observed not to form flares (*30*) or picocavities.

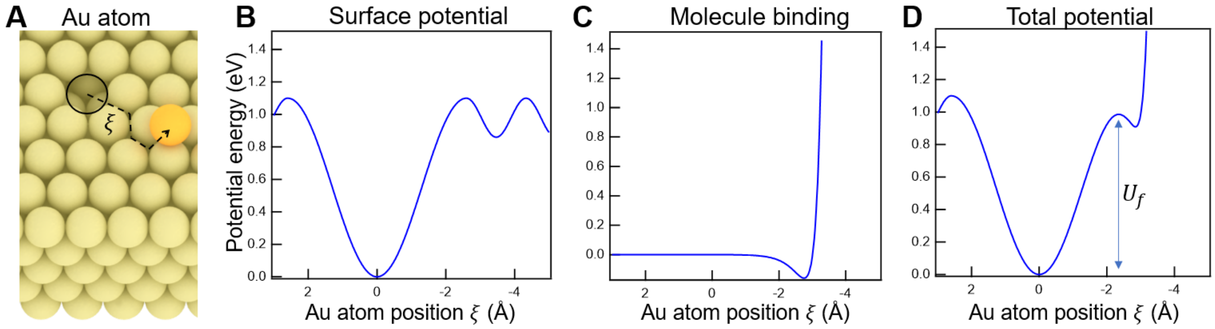

Fig. S20. Potential for Au atom pulling out of facet. (**A**) schematic for Au atom (black circle) lifting out of pit onto facet surface (dashed arrow, trajectory ξ). (**B**) Potential energy for atom along trajectory with no additional molecule. (**C**) Potential energy for Au adatom as approaches molecule situated at -4.5 Å, showing formation of coordination bond (for BPT here). (**D**) Total potential energy showing size of barrier for adatom to escape pit to coordination bond site, U_f~1 eV.

Total Au atom potential: We now combine the intensity-dependent optical force term derived in **Eq. S14** together with the original potential for moving a Au atom from inside the facet to its adatom position, to

give **Model 2**. Inside the facet the Au-Au coordination number is 9, but this reduces to 2 as it pulls out of its pit position (black circle, **Fig. S20A**).

The potential energy as the Au atom is pulled out of the facet onto the surface follows **Fig. S20B**, showing the $U_f \sim 1$ eV size of the barrier normally preventing any adatoms escaping onto a flat facet at room temperature. Note that step edges and defects do not change this unless they directly reduce the initial coordination number (so only for Au atoms at vertices and edges). Using DFT to model the energy of the Au-molecule energy as a function of separation (**Fig. S20C**) shows the coordination bond whose energy depth varies from 160 meV for BPT (shown here) to 1 eV for MPy. These can combined into a static potential before light is then applied to the system (**Fig. S20D**).

Our experiments (see main text) imply that (i) adatoms (giving picocavities) or adlayers (giving flares) are produced thermally over (ii) an energy barrier which for increasing light intensity is progressively reduced in height. This leads to a model for the probability of creating a picocavity or a flare

$$P = P_0 \exp\{-U_f(I)/k_B T\}$$

where the forward barrier $U_f(I)$ can then be extracted from the experimental data. We find that $U_f(I)$ lies on a universal curve for different molecules (**Fig. S21**) if it is plotted as a function of $U_f(\alpha_t I)$ where $\alpha_t \propto q/\mathbf{p}$ is the local polarizability extracted from the DFT (see above) for the tip atom on the molecule.

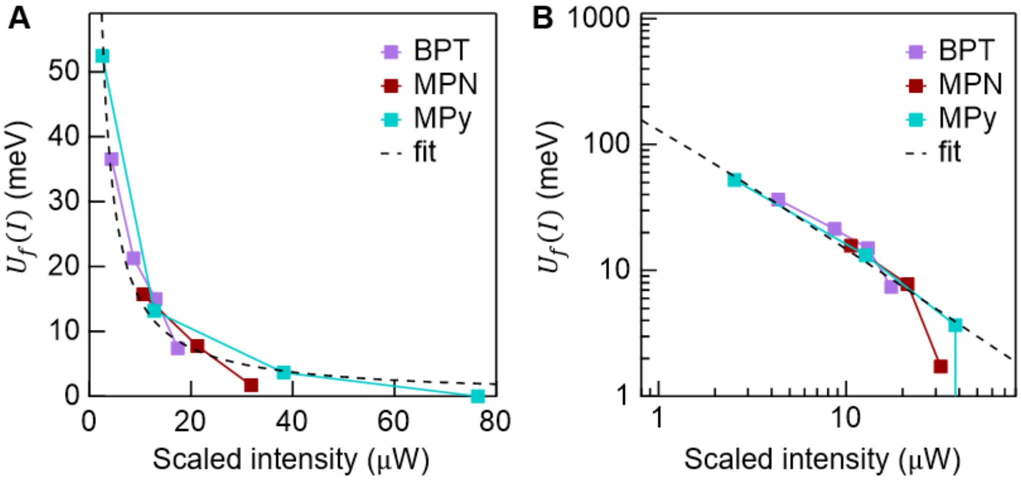

Fig. S21. Universal curve for barrier height *vs* scaled laser intensity ($I' = \alpha_t I$). A, $U_f(I')$ on a linear scale and (**B**) on a log-log scale. Dashed line is the fit given in **Eq. S15**.

This universal curve is fit rather well by

$$U_f = \frac{U_f^0}{1 + I/I_t} \qquad \text{(S15)}$$

shown as the dashed curve, which as needed retains the initial $U_f(I=0) = U_f^0 \sim 1$ eV barrier with the light off, but rapidly reduces with light intensity. For picocavities to become likely to excite thermally (at $U_f(I_c) = k_B T$) then $I_c = I_t U_f^0/k_B T \sim 40 I_t$ at room temperature.
Even at the highest light intensity it is clear that this barrier does not disappear, which matches what is seen in the experiments with still a residual thermal barrier even at the largest powers. While this fit function is only approximate, it gives the main features here, and in particular cannot be replicated by any laser-heating model. Considering optical forces alone, which would tilt the potential seen in **Fig. S20D** to gradually reduce the barrier, the problems are that (i) the optical forces alone are too weak to overcome

the high initial barrier, and (ii) after the barrier becomes low enough for thermal excitation over it, at slightly higher intensities it would then disappear, giving instant picocavities which is not what is seen in experiment.

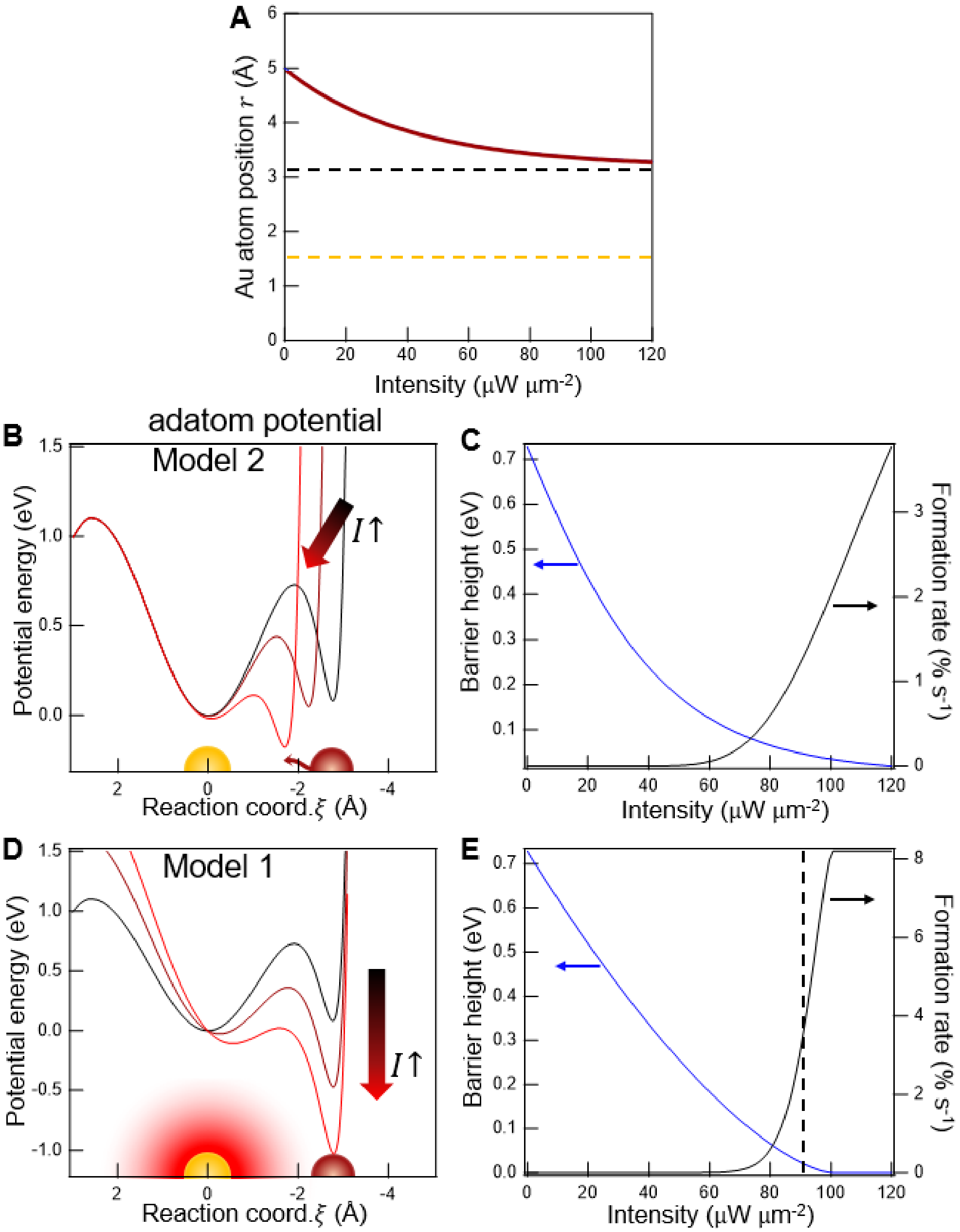

Fig. S22. Models for adatom potential under irradiation. (**A**) Position of adatom relative to centre of molecule tip atom *vs* laser power used, which saturates at the sum of the ionic radii. (**B**) Total Au potential as adatom approaches molecule tip atom (relative to Au atom position) and (**C**) corresponding extracted barrier height and resulting picocavity formation rate at T=300K. (**D**) Total potential using scaled up optical force which tilts the adatom potential and (**E**) corresponding barrier height which decreases much faster than in experiment resulting in a sharp turn-on of picocavity formation that is not seen.

Instead we suggest that what happens is that when the light is applied, the additional force brings the adatom slightly closer to the molecule. This slightly decreases the barrier, and allows the adatom to approach slightly more (**Fig. S22B**), enhancing tip polarizability and thus reducing the barrier, to bring the adatom yet closer, in a positive feedback. What stops this ultimately is the overlap of the electron clouds of Au adatom and molecule tip atom (by Pauli exclusion). We thus assume that there is a closest distance of approach which is possible, irrespective of power (modelled as a saturation, see **Fig. S22A**). The resulting barrier energy to adatoms escaping from within the Au surface then decreases as laser intensity increases (**Fig. S22C**), which yields a picocavity formation rate in good agreement with experiment. In the fits presented in the main text, we use the **Eq. S15** to extract values of the critical intensity. In addition, the reduction of barrier when illumination is present would explain why the decay of picocavities tracks the optical power in exactly the same way as their creation rate (**Fig. 3**). No other model can explain this. If we instead use an optical force (Model 1) which simply scales with the field gradient ($F \propto \nabla\mathcal{E}^2$), and is set here artificially strong enough to overcome the Au binding U_f^0 (**Fig. S22D**), then there is a sudden turn on of picocavities when the barrier is overcome (dashed line **Fig. S22E**), which is not what is observed. In addition, this model would also suggest that the reverse barrier once picocavities have been created is equally large, so picocavities would never be observed to decay (again contradicting experiment).

Supplementary Note S9. Summary of evidence

We collect here a summary of the different phenomena, and how they support the field-plucking model. A list of the experimental features observed is:

E1) The picocavity generation rate increases with laser power above threshold, then saturates (instead of exponential growth).

E2) Histogram of creation times for picocavity decays exponentially, implying a fixed probability of creation.

E3) Different molecules have characteristic picocavity generation rates (per local intensity). A stronger metal-molecule coordination bond gives a lower laser threshold (easier to create), and lower decay rate (harder to destroy).

E4) Picocavity decay rates depend on laser power exactly as creation rates. More than two-thirds of picocavities are less stable, decaying ten-fold faster.

E5) Picocavities are produced independently (one does not cascade many, or turn off all). There is only very slight statistical evidence that if a picocavity is observed, it gives an increased likelihood to see a second one in the same scan.

E6) Picocavity SERS lines fluctuate over ~1s (at rates 4cm^{-1}/s for BPTCN for 25μW).(*21*)

E7) Picocavity creation rates are found to be much slower on (100) Au facets.(*56*)

E8) Picocavities are much harder to create at low temperature above threshold, and much more stable to decay.

E9) Picocavity SERS rise times are 60μs, decay times around 80μs.(*57*)

The field-plucking model explains:

E1) Light intensity needs to be high enough to reduce the barrier to thermal energy scales in order to start picocavity formation. But this barrier does not completely disappear due to limits on approach of electronic orbitals.

E2) Thermal excitation over an optically-reduced barrier explains this.

E3) Rate of optically-induced barrier reduction depends on local polarizability at the molecule tip. For alkanethiols the polarizability is very small, and few picocavities are observed (though their observation is challenging since their Raman cross sections are also small - flares which do not depend on the Raman cross section are however also minimal).

E4) With light off, the energy well for picocavities is also much higher than kT. The system needs optical reduction of the barrier to allow the adatom to return into the facet. Adatoms can thus be trapped on the suface.
E5) Whichever molecule tip has the largest local polarizability will act first, depending on nearby ions, the exact registration with atoms on facet, and likely other factors sensing the local atomic environment.
E6) Thermal diffusion inside the coordination-bond potential well allows Brownian motion of the adatom (exciting different thermal superpositions of vibrational sub-levels). Illuminating with light reduces this barrier, softens the adatom potential well, and increases diffusion rates.
E7) The initial barrier height is ~2 eV larger on Au(100) (*23*, *56*, *58*) and thus requires much larger laser powers for optical plucking.
E8) At low temperature, a much larger laser power is needed to reduce the barrier height sufficiently to allow thermal excitation of picocavities. This would not be the case for any heating effect (since to get to T=10,000K it is not relevant whether starting at T=10K or T=300K). At T=10K, once formed, picocavities are stable for extremely long times if the light is off, as the full 1 eV barrier is restored.

Supplementary Note S10. Alternative models
We explore several other models to try to explain these observations.

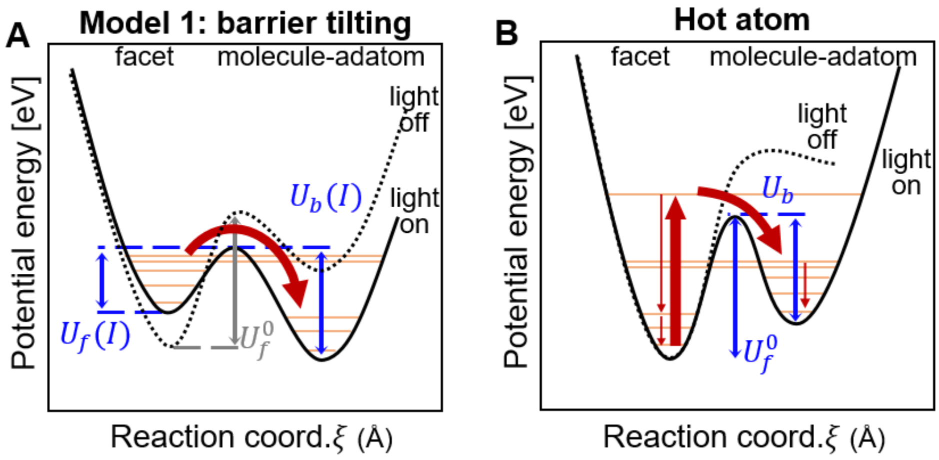

Fig. S23. Alternative models for picocavity and flare generation. (**A**) Optical forces tilting the initial barrier. (**B**) Hot atom model in which plasmon energy is given to an individual Au atom at the surface pushing it towards adatom where it coordination bonds (if $\boldsymbol{U_f^0 < \hbar\omega_p}$).

A) Hot-atom ejection, where the Au atom is given the entire plasmon energy to get over the barrier U_f^0. This is similar to the 'hot-electron model', in which the plasmon decays giving its energy to a single electron, and we now envisage this same energy is given to a single surface Au atom. If $U_f^0 > \hbar\omega_p$ (**Fig. S23B**) then it is not clear how thermal energies would be sufficient to supply the remaining energy. Atom tunnelling is found to be negligible since for an atom of wavevector k, $ka{\sim}a\left(2mU_f^0\right)^{1/2}/\hbar{\sim}145$ so that a tunnelling probability $\propto \exp(-ka)$ is too small to play any role. If $U_f^0 < \hbar\omega_p$ then it is hard to see why the molecule itself would make any difference in producing different picocavity generation rates.
This model also does not fit the temperature dependence, nor gives any understanding of how picocavities could decay since a similar barrier has to be overcome (U_b^0), while it is not clear how the hot

atom forces would attract it back now into the facet (there is no equivalent of hot hole emission for atoms).

B) Electronic Raman scattering (ERS) electronic ejection, where the driven Raman process acting on electrons in the Fermi sea generates a kick against a single Au atom. A simple estimate for the momentum change between the two electronic states separated by the plasmon energy is $p = \hbar\Delta k = \sqrt{2m(E_F + \hbar\omega)} - \sqrt{2mE_F}$. If this momentum change acts over typical scattering times of 10fs it would thus give forces of ~10pN/photon, again too small to have any effect here.

C) Multiphoton absorption, where excitation of the Au atom in its vibrational well would be followed by subsequent excitation to get it over U_f^0. Recently we have shown that multi-photon absorption in molecules in these gaps is strongly enhanced.(*59*) It is difficult however to see why the molecule at the surface would influence the excitation of the Au atom to high energies, while sensible estimates of the number of plasmons absorbed per µW incident shows that at 5 µW there would be 30 ps between absorption events, long enough for all vibrational energy injected into a single Au atom to have decayed. There is also little evidence of a multiphoton power dependence in the signatures described.

D) Optomechanical forces, where vibrational driving of the Au-Au bonds leads to a parametric instability that drives out a Au adatom. The natural oscillation frequency of this Au-Au facet bond assuming a parabolic potential so that $\nu = \sqrt{k/m}$ and with $U_f^0 \sim \frac{1}{2}ka^2$ gives an estimate for $\hbar\nu$ = 7 meV or 60 cm^{-1}. There are few experiments which have attempted to look at Au-Au vibrational frequencies, mostly in heavy-metal dimer (*60*) or trimer molecules,(*61*) and they give reports from $\hbar\nu$ =10-100 cm^{-1} in various molecular complexes, with none seen so far for bulk Au. Thermal population of these low energy vibrations is insufficient to reach the U_f^0 energy barrier.

REFERENCES AND NOTES

1. B. S. Hoener, S. R. Kirchner, T. S. Heiderscheit, S. S. E. Collins, W. S. Chang, S. Link, C. F. Landes, Plasmonic sensing and control of single-nanoparticle electrochemistry. *Chem* **4**, 1560–1585 (2018).

2. L. Zhou, D. F. Swearer, C. Zhang, H. Robatjazi, H. Zhao, L. Henderson, L. Dong, P. Christopher, E. A. Carter, P. Nordlander, N. J. Halas, Quantifying hot carrier and thermal contributions in plasmonic photocatalysis. *Science* **362**, 69–72 (2018).

3. G. Li, R. Zhu, Y. Yang, Polymer solar cells. *Nat. Photonics* **6**, 153–161 (2012).

4. B. Sepúlveda, P. C. Angelomé, L. M. Lechuga, L. M. Liz-Marzán, LSPR-based nanobiosensors. *Nano Today* **4**, 244–251 (2009).

5. P. K. Jain, X. Huang, I. H. El-Sayed, M. A. El-Sayed, Noble metals on the nanoscale: Optical and photothermal properties and some applications in imaging, sensing, biology, and medicine. *Acc. Chem. Res.* **41**, 1578–1586 (2008).

6. M. Galperin, Photonics and spectroscopy in nanojunctions: A theoretical insight. *Chem. Soc. Rev.* **46**, 4000–4019 (2017).

7. N. Xin, J. Guan, C. Zhou, X. Chen, C. Gu, Y. Li, M. A. Ratner, A. Nitzan, J. F. Stoddart, X. Guo, Concepts in the design and engineering of single-molecule electronic devices. *Nat. Rev. Phys.* **1**, 211–230 (2019).

8. P. Gehring, J. M. Thijssen, H. S. J. van der Zant, Single-molecule quantum-transport phenomena in break junctions. *Nat. Rev. Phys.* **1**, 381–396 (2019).

9. A. R. Rocha, V. M. García-suárez, S. W. Bailey, C. J. Lambert, J. Ferrer, S. Sanvito, Towards molecular spintronics. *Nat. Mater.* **4**, 335–339 (2005).

10. L. Bogani, W. Wernsdorfer, Molecular spintronics using single-molecule magnets. *Nat. Mater.* **7**, 179–186 (2008).

11. S. Sanvito, Molecular spintronics. *Chem. Soc. Rev.* **40**, 3336–3355 (2011).

12. S. Schlücker, Surface-enhanced raman spectroscopy: Concepts and chemical applications. *Angew. Chemie Int. Ed.* **53**, 4756–4795 (2014).

13. M. Barbry, P. Koval, F. Marchesin, R. Esteban, A. G. Borisov, J. Aizpurua, D. Sánchez-Portal, Atomistic near-field nanoplasmonics: Reaching atomic-scale resolution in nanooptics. *Nano Lett.* **15**, 3410–3419 (2015).

14. S. Trautmann, J. Aizpurua, I. Götz, A. Undisz, J. Dellith, H. Schneidewind, M. Rettenmayr, V. Deckert, A classical description of subnanometer resolution by atomic features in metallic structures. *Nanoscale* **9**, 391–401 (2017).

15. M. Urbieta, M. Barbry, Y. Zhang, P. Koval, D. Sánchez-Portal, N. Zabala, J. Aizpurua, Atomic-scale lightning rod effect in plasmonic picocavities: A classical view to a quantum effect. *ACS Nano* **12**, 585–595 (2018).

16. R. Zhang, Y. Zhang, Z. C. Dong, S. Jiang, C. Zhang, L. G. Chen, L. Zhang, Y. Liao, J. Aizpurua, Y. Luo, J. L. Yang, J. G. Hou, Chemical mapping of a single molecule by plasmon-enhanced Raman scattering. *Nature* **498**, 82–86 (2013).

17. J. Lee, K. T. Crampton, N. Tallarida, V. A. Apkarian, Visualizing vibrational normal modes of a single molecule with atomically confined light. *Nature* **568**, 78–82 (2019).

18. M. Richard-Lacroix, V. Deckert, Direct molecular-level near-field plasmon and temperature assessment in a single plasmonic hotspot. *Light Sci. Appl.* **9**, 35 (2020).

19. F. Benz, M. K. Schmidt, A. Dreismann, R. Chikkaraddy, Y. Zhang, A. Demetriadou, C. Carnegie, H. Ohadi, B. de Nijs, R. Esteban, J. Aizpurua, J. J. Baumberg, Single-molecule optomechanics in "picocavities". *Science* **354**, 726–729 (2016).

20. H.-H. Shin, G. J. Yeon, H.-K. Choi, S.-M. Park, K. S. Lee, Z. H. Kim, Frequency-domain proof of the existence of atomic-scale SERS hot-spots. *Nano Lett.* **18**, 262–271 (2018).

21. C. Carnegie, J. Griffiths, B. de Nijs, C. Readman, R. Chikkaraddy, W. M. Deacon, Y. Zhang, I. Szabó, E. Rosta, J. Aizpurua, J. J. Baumberg, Room-temperature optical picocavities below 1 nm^3 accessing single-atom geometries. *J. Phys. Chem. Lett.* **9**, 7146–7151 (2018).

22. J. Huang, D.-B. Grys, J. Griffiths, B. de Nijs, M. Kamp, Q. Lin, J. J. Baumberg, Tracking interfacial single-molecule pH and binding dynamics via vibrational spectroscopy. *Sci. Adv.* **7**, eabg1790 (2021).

23. L. Vitos, A. V. Ruban, H. L. Skriver, J. Kollár, The surface energy of metals. *Surf. Sci.* **411**, 186–202 (1998).

24. J. Takano, O. Takai, Y. Kogure, M. Doyama, Simulation of atomic-scale surface migration in homoepitaxial growth using embedded-atom method potentials for gold. *Thin Solid Films* **318**, 52–56 (1998).

25. Y. Liu, V. Ozolins, Self-assembled monolayers on Au(111): Structure, energetics, and mechanism of reconstruction lifting. *J. Phys. Chem. C* **116**, 4738–4747 (2012).

26. D. Thompson, J. Liao, M. Nolan, A. J. Quinn, C. A. Nijhuis, C. O'Dwyer, P. N. Nirmalraj, C. Schönenberger, M. Calame, Formation mechanism of metal–molecule–metal junctions: Molecule-assisted migration on metal defects. *J. Phys. Chem. C* **119**, 19438–19451 (2015).

27. T.-S. Lin, Y.-W. Chung, Measurement of the activation energy for surface diffusion in gold by scanning tunneling microscopy. *Surf. Sci.* **207**, 539–546 (1989).

28. J. J. Baumberg, J. Aizpurua, M. H. Mikkelsen, D. R. Smith, Extreme nanophotonics from ultrathin metallic gaps. *Nat. Mater.* **18**, 668–678 (2019).

29. J. Langer, D. Jimenez de Aberasturi, J. Aizpurua, R. A. Alvarez-Puebla, B. Auguié, J. J. Baumberg, G. C. Bazan, S. E. J. Bell, A. Boisen, A. G. Brolo, J. Choo, D. Cialla-May, V. Deckert, L. Fabris, K. Faulds, F. J. García de Abajo, R. Goodacre, D. Graham, A. J. Haes, C. L. Haynes, C. Huck, T. Itoh, M. Käll, J. Kneipp, N. A. Kotov, H. Kuang, E. C. Le Ru, H. K. Lee, J.-F. Li, X. Y. Ling, S. A. Maier, T. Mayerhöfer, M. Moskovits, K. Murakoshi, J.-M. Nam, S. Nie, Y. Ozaki, I. Pastoriza-Santos, J. Perez-Juste, J. Popp, A. Pucci, S. Reich, B. Ren, G. C. Schatz, T. Shegai, S. Schlücker,

L.-L. Tay, K. G. Thomas, Z.-Q. Tian, R. P. Van Duyne, T. Vo-Dinh, Y. Wang, K. A. Willets, C. Xu, H. Xu, Y. Xu, Y. S. Yamamoto, B. Zhao, L. M. Liz-Marzán, Present and future of surface-enhanced raman scattering. *ACS Nano* **14**, 28–117 (2020).

30. C. Carnegie, M. Urbieta, R. Chikkaraddy, B. de Nijs, J. Griffiths, W. M. Deacon, M. Kamp, N. Zabala, J. Aizpurua, J. J. Baumberg, Flickering nanometre-scale disorder in a crystal lattice tracked by plasmonic flare light emission. *Nat. Commun.* **11**, 682 (2020).

31. F. Benz, B. de Nijs, C. Tserkezis, R. Chikkaraddy, D. O. Sigle, L. Pukenas, S. D. Evans, J. Aizpurua, J. J. Baumberg, Generalized circuit model for coupled plasmonic systems. *Opt. Express* **23**, 33255–33269 (2015).

32. J. Griffiths, T. Földes, B. de Nijs, R. Chikkaraddy, D. Wright, W. M. Deacon, D. Berta, C. Readman, D. B. Grys, E. Rosta, J. J. Baumberg, Resolving sub-angstrom ambient motion through reconstruction from vibrational spectra. *Nat. Commun.* **12**, 6759 (2021).

33. J. Griffiths, B. de Nijs, R. Chikkaraddy, J. J. Baumberg, Locating single-atom optical picocavities using wavelength-multiplexed raman scattering. *ACS Photonics* **8**, 2868–2875 (2021).

34. Y.-Y. Cai, E. Sung, R. Zhang, L. J. Tauzin, J. G. Liu, B. Ostovar, Y. Zhang, W.-S. Chang, P. Nordlander, S. Link, Anti-stokes emission from hot carriers in gold nanorods. *Nano Lett.* **19**, 1067–1073 (2019).

35. B. de Nijs, R. W. Bowman, L. O. Herrmann, F. Benz, S. J. Barrow, J. Mertens, D. O. Sigle, R. Chikkaraddy, A. Eiden, A. Ferrari, O. A. Scherman, J. J. Baumberg, Unfolding the contents of sub-nm plasmonic gaps using normalising plasmon resonance spectroscopy. *Faraday Discuss.* **178**, 185–193 (2015).

36. J. N. Sweet, The spectral similarity scale and its application to the classification of hyperspectral remote sensing data, in *IEEE Workshop on Advances in Techniques for Analysis of Remotely Sensed Data* (IEEE, 2003), pp. 92–99.

37. N. Kongsuwan, A. Demetriadou, M. Horton, R. Chikkaraddy, J. J. Baumberg, O. Hess, Plasmonic nanocavity modes: From near-field to far-field radiation. *ACS Photonics* **7**, 463–471 (2020).

38. C. M. Teodorescu, Image molecular dipoles in surface enhanced Raman scattering. *Phys. Chem. Chem. Phys.* **17**, 21302–21314 (2015).

39. W. Du, T. Wang, H.-S. Chu, L. Wu, R. Liu, S. Sun, W. K. Phua, L. Wang, N. Tomczak, C. A. Nijhuis, On-chip molecular electronic plasmon sources based on self-assembled monolayer tunnel junctions. *Nat. Photonics* **10**, 274–280 (2016).

40. T. Wang, W. Du, N. Tomczak, L. Wang, C. A. Nijhuis, In operando characterization and control over intermittent light emission from molecular tunnel junctions via molecular backbone rigidity. *Adv. Sci.* **6**, 1900390 (2019).

41. H. Häkkinen, The gold–sulfur interface at the nanoscale. *Nat. Chem.* **4**, 443–455 (2012).

42. BBI Solutions, *Diagnostic Gold Colloid*; www.bbisolutions.com/en/reagents/gold-colloid.

43. F. Benz, R. Chikkaraddy, A. Salmon, H. Ohadi, B. de Nijs, J. Mertens, C. Carnegie, R. W. Bowman, J. J. Baumberg, SERS of individual nanoparticles on a mirror: Size does matter, but so does shape. *J. Phys. Chem. Lett.* **7**, 2264–2269 (2016).

44. G. Baffou, R. Quidant, F. J. García de Abajo, Nanoscale control of optical heating in complex plasmonic systems. *ACS Nano* **4**, 709–716 (2010).

45. D. Wright, Q. Lin, D. Berta, T. Földes, A. Wagner, J. Griffiths, C. Readman, E. Rosta, E. Reisner, J. J. Baumberg, Mechanistic study of an immobilized molecular electrocatalyst by in situ gap-plasmon-assisted spectro-electrochemistry. *Nat. Catal.* **4**, 157–163 (2021).

46. A. Allouche, Software news and updates Gabedit—A graphical user interface for computational chemistry softwares. *J. Comput. Chem.* **32**, 174–182 (2012).

47. M. J. Frisch, G. W. Trucks, H. B. Schlegel, G. E. Scuseria, M. A. Robb, J. R. Cheeseman, G. Scalmani, V. Barone, B. Mennucci, G. A. Petersson, H. Nakatsuji, M. Caricato, X. Li, H. P. Hratchian, A. F. Izmaylov, J. Bloino, G. Zheng, J. L. Sonnenberg, M. Hada, M. Ehara, K. Toyota, R. Fukuda, J. Hasegawa, M. Ishida, T. Nakajima, Y. Honda, O. Kitao, H. Nakai, T. Vreven, J. A. Montgomery, J. E. Peralta, F. Ogliaro, M. Bearpark, J. J. Heyd, E. Brothers, K. N. Kudin, V. N.

Staroverov, R. Kobayashi, J. Normand, K. Raghavachari, A. Rendell, J. C. Burant, S. S. Iyengar, J. Tomasi, M. Cossi, N. Rega, J. M. Millam, M. Klene, J. E. Knox, J. B. Cross, V. Bakken, C. Adamo, J. Jaramillo, R. Gomperts, R. E. Stratmann, O. Yazyev, A. J. Austin, R. Cammi, C. Pomelli, J. W. Ochterski, R. L. Martin, K. Morokuma, V. G. Zakrzewski, G. A. Voth, P. Salvador, J. J. Dannenberg, S. Dapprich, A. D. Daniels, Ö. Farkas, J. B. Foresman, J. V Ortiz, J. Cioslowski, D. J. Fox, Gaussian 09 Revision E (2009).

48. T. Giannakopoulos, PyAudioAnalysis: An open-source python library for audio signal analysis. *PLOS ONE* **10**, e0144610 (2015).

49. A. Moroz, Depolarization field of spheroidal particles. *J. Opt. Soc. Am. B.* **26**, 517 (2009).

50. E. M. Purcell, D. J. Morin, *Electricity and Magnetism* (Cambridge Univ. Press, ed. 3, 2013).

51. F. D. Nunes, T. C. Vasconcelos, M. Bezerra, J. Weiner, Electromagnetic energy density in dispersive and dissipative media. *J. Opt. Soc. Am. B.* **28**, 1544–1552 (2011).

52. A. Ashkin, Optical trapping and manipulation of neutral particles using lasers. *Proc. Natl. Acad. Sci.* **94**, 4853–4860 (1997).

53. D.-B. Grys, B. de Nijs, A. R. Salmon, J. Huang, W. Wang, W.-H. Chen, O. A. Scherman, J. J. Baumberg, Citrate coordination and bridging of gold nanoparticles: The role of gold adatoms in AuNP aging. *ACS Nano* **14**, 8689–8696 (2020).

54. M. Giesen, Step and island dynamics at solid/vacuum and solid/liquid interfaces. *Prog. Surf. Sci.* **68**, 1–154 (2001).

55. N. V. Smith, C. T. Chen, M. Weinert, Distance of the image plane from metal surfaces. *Phys. Rev. B.* **40**, 7565–7573 (1989).

56. A. Xomalis, R. Chikkaraddy, E. Oksenberg, I. Shlesinger, J. Huang, E. C. Garnett, A. F. Koenderink, J. J. Baumberg, Controlling optically driven atomic migration using crystal-facet control in plasmonic nanocavities. *ACS Nano* **14**, 10562–10568 (2020).

57. M. J. Horton, O. S. Ojambati, R. Chikkaraddy, W. M. Deacon, N. Kongsuwan, A. Demetriadou, O. Hess, J. J. Baumberg, Nanoscopy through a plasmonic nanolens. *Proc. Natl. Acad. Sci.* **117**, 2275–2281 (2020).

58. M. Grzelczak, J. Pérez-Juste, P. Mulvaney, L. M. Liz-Marzán, Shape control in gold nanoparticle synthesis. *Chem. Soc. Rev.* **37**, 1783–1791 (2008).

59. O. S. Ojambati, R. Chikkaraddy, W. M. Deacon, J. Huang, D. Wright, J. J. Baumberg, Efficient generation of two-photon excited phosphorescence from molecules in plasmonic nanocavities. *Nano Lett.* **20**, 4653–4658 (2020).

60. C. Latouche, Y.-R. Lin, Y. Tobon, E. Furet, J.-Y. Saillard, C.-W. Liu, A. Boucekkine, Au–Au chemical bonding induced by UV irradiation of dinuclear gold(I) complexes: A computational study with experimental evidence. *Phys. Chem. Chem. Phys.* **16**, 25840–25845 (2014).

61. H. Kuramochi, S. Takeuchi, M. Iwamura, K. Nozaki, T. Tahara, Tracking photoinduced Au–Au bond formation through transient terahertz vibrations observed by femtosecond time-domain raman spectroscopy. *J. Am. Chem. Soc.* **141**, 19296–19303 (2019).
